# Intrinsic DNA curvature in trypanosomes

**DOI:** 10.1186/s13104-017-2908-y

**Published:** 2017-11-09

**Authors:** Pablo Smircich, Najib M. El-Sayed, Beatriz Garat

**Affiliations:** 1 Laboratorio de Interacciones Moleculares, Facultad de Ciencias, Universidad de la Republica, 11400 Montevideo, Uruguay; 2 Departamento de Genética, Facultad de Medicina, Universidad de la Republica, 11800 Montevideo, Uruguay; 3 Department of Cell Biology and Molecular Genetics and Center for Bioinformatics and Computational Biology, University of Maryland College Park, College Park, MD 20742 USA

**Keywords:** *Trypanosoma*, *cruzi*, *brucei*, DNA intrinsic curvature, Transcription, VSG, Base J

## Abstract

**Background:**

*Trypanosoma cruzi* and *Trypanosoma brucei* are protozoan parasites causing Chagas disease and African sleeping sickness, displaying unique features of cellular and molecular biology. Remarkably, no canonical signals for RNA polymerase II promoters, which drive protein coding genes transcription, have been identified so far. The secondary structure of DNA has long been recognized as a signal in biological processes and more recently, its involvement in transcription initiation in *Leishmania* was proposed. In order to study whether this feature is conserved in trypanosomatids, we undertook a genome wide search for intrinsic DNA curvature in *T. cruzi* and *T. brucei*.

**Results:**

Using a region integrated intrinsic curvature (RIIC) scoring that we previously developed, a non-random distribution of sequence-dependent curvature was observed. High RIIC scores were found to be significantly correlated with transcription start sites in *T. cruzi*, which have been mapped in divergent switch regions, whereas in *T. brucei*, the high RIIC scores correlated with sites that have been involved not only in RNA polymerase II initiation but also in termination. In addition, we observed regions with high RIIC score presenting in-phase tracts of Adenines, in the subtelomeric regions of the *T. brucei* chromosomes that harbor the variable surface glycoproteins genes.

**Conclusions:**

In both *T. cruzi* and *T. brucei* genomes, a link between DNA conformational signals and gene expression was found. High sequence dependent curvature is associated with transcriptional regulation regions. High intrinsic curvature also occurs at the *T. brucei* chromosome subtelomeric regions where the recombination processes involved in the evasion of the immune host system take place. These findings underscore the relevance of indirect DNA readout in these ancient eukaryotes.

**Electronic supplementary material:**

The online version of this article (10.1186/s13104-017-2908-y) contains supplementary material, which is available to authorized users.

## Background

*Trypanosoma cruzi* and *Trypanosoma brucei* (family Trypanosomatidae, order Kinetoplastida) are flagellated protozoan parasites that cause Chagas disease and African sleeping sickness, respectively. They infect the poorest rural populations in developing countries in tropical and subtropical regions leading to tens of thousands of human deaths every year [1].

Although trypanosomes share many characteristics at the molecular and biochemical levels, they exhibit different life cycles. *T. cruzi* has a complex life cycle alternating between two extracellular forms in the triatomine insect: the epimastigote and the infective metacyclic trypomastigote, and two forms in the mammalian host: the intracellular amastigote and the infective bloodstream trypomastigote. In contrast, *T. brucei* is exclusively extracellular, alternating between the procyclic form in the tsetse fly and the bloodstream form in the mammalian host [2]. In order to evade the immune system, *T. brucei* forms a dense coat of variant surface glycoproteins (VSG) that are expressed one at a time from telomeric expression sites and are derived from a repertoire of up to 2000 genes [3, 4]. The high expression of a single VSG gene is carried out by an RNA polymerase I (RNAPI) capable of high transcription initiation rates [5]. This mRNA synthesis property by RNAPI is one of the molecular characteristics that distinguish this parasite from other eukaryotic organisms. Recently, we determined that the eukaryotic conserved intrinsic curvature, which is a characteristic of the RNAPI core promoters, is present not only at the rDNA loci of *T. brucei*, *T. cruzi* and *Leishmania* but also within RNAPI promoters involved in transcribing protein coding genes in *T. brucei* (VSG and procyclins) [6].

Trypanosomes have additional unique gene organization and expression features, including the organization of genes in directional clusters, the constitutive transcription of large polycistronic gene units, the amplification of genes in response to environmental stimuli, the *trans*-splicing of mRNAs, the extensive editing of mitochondrial transcripts [7, 8] and the dependence on post transcriptional regulation mechanisms to coordinate gene expression [2, 9]. Subtelomeric regions are composed of variable repetitive elements and contain genes involved in antigenic variation in *T. brucei* or genes encoding surface antigens in *T. cruz*i [10, 11]. The hyper-modified base J (β-d-Glucopyranosyloxymethyluracil), predominantly present in repetitive DNA sequences in telomeres and subtelomeres of trypanosomatids, has been more recently localized in RNA polymerase II transcription initiation and termination sites [12]. Canonical signals for RNA polymerase II promoters have only been described for the genes encoding the spliced leader (SL), a small RNA added by *trans*-splicing to all the protein coding genes [13]. Nonetheless, transcription starting sites (TSS) [14, 15] and histone variants implicated in the initiation process [16, 17] have been described mainly at the strand switch regions (SSRs) that separate the heads of the polycistronic gene units, named divergent SSRs (DSSRs). On the contrary, the SSRs that separate the tails of the polycistronic gene units, named convergent SSRs (CSSRs), have been shown to preferentially contain sites of transcription termination as well as polymerase III transcribed tRNA genes [15, 18]. Transcription also terminates and initiates at internal TSSs, where transcription of an upstream polycistronic unit terminates and transcription of a downstream polycistronic unit on the same strand initiates [17, 19, 20]. A bias in poly-dinucleotides abundance has also been reported for those regions [21]. A link between transcription and DNA replication has been recently described in *T. brucei* [22]. In spite of the important insights achieved (reviewed in [23]), the molecular signals underlying these processes remain mostly under investigation [24, 25].

Intrinsically curved DNA structures are often found around origins of DNA replication, DNA recombination loci and in regions that regulate transcription. In eukaryotes, this feature is common not only to the promoters of genes transcribed by RNA polymerase I, but also to many promoters of RNA polymerase II (reviewed in [26]). We have previously reported an association of transcription start sites with regions of high regional integrated intrinsic curvature (RIIC) score in *Leishmania* [27]. Though differences between trypanosomes and *Leishmania*, due to different base composition content and derived intrinsic curvature (IC) may exist, here we studied whether high RIIC regions could also be associated to a biological phenomenon in *T. cruzi* and *T. brucei*. We found that high RIIC scores were indeed associated with regions involved in transcriptional regulation, such as DSSRs in *T. cruzi* and regions enriched in base J in *T. brucei*. A concentration of high curvature regions in the *T. brucei* subtelomeres, overlapping regions of silent VSGs, was also observed, and a canonical signal for DNA bending consisting on A tracts repeated in phase was discovered therein. Our findings suggest a link between DNA conformational signals and gene expression in trypanosomes.

## Results

**Fig. 1:**
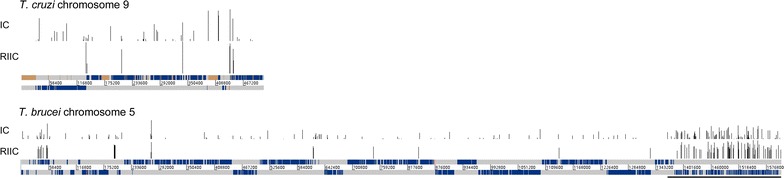
Graphical representation of sequence dependent curvature. A schematic representation of the indicated trypanosomatid chromosomes are presented. Upper panel: Bar plots of chromosome positions with an IC value greater than 13  per helical turn. Middle panel: bar plots of chromosome positions with an RIIC value greater than the selected cutoff. Lower panel: both DNA strands are depicted in grey, overlaid with CDS features shown in blue. Features labeled as ncRNA, snRNA or snoRNAs are shown in green. tRNAs are shown in red. Assembly gaps are shown in brown. For *T. brucei* chromosome 5 subtelomeric VSG clusters are underlined

Since we have previously shown that sequence-dependent DNA curvature may have a role in transcription initiation in *Leishmania* [27], we investigated its genomic distribution in the related trypanosomatid parasites *T. cruzi* and *T. brucei*. The *T. cruzi* CL Brener Esmeraldo-like haplotype [11] and the *T. brucei* 11 Megabase-sized chromosomes [28] were used. Among the various programs to determine sequence dependent DNA curvature in silico, we selected the bend.it calculation because it is based on multiple dinucleotide and trinucleotide models [29]. The output consists on predicted curvature angles per helical turn of the double helix (degrees/hel. turn) for each nucleotide calculated on short DNA windows which slide along each chromosome sequence. An initial DNA curvature analysis was carried on both genomes as previously described [30]. Concurrently, we analyzed genomic curvatures using the RIIC scoring function we previously developed, that specifically finds regions that accumulate curved sites [27]. A sample distribution of regions of high sequence-dependent curvature using both approaches for *T. cruzi* chromosome 9 and *T. brucei* chromosome 5 is shown in Fig. 1 (see Additional file 3: Figure S1 and Additional file 4: Figure S2 for all the *T. cruzi* and *T.* *brucei* chromosomes, respectively. Wiggle format files containing the positions and magnitude of plotted intrinsic curvature are provided as Additional file 2). While in *T. cruzi* the high RIIC regions are apparently associated with strand switch regions, this observation is not so obvious for *T. brucei*. Nevertheless, it seems that also in *T. brucei*, regions of high curvature are not randomly distributed. Indeed, a clear concentration of regions of high curvature can readily be observed at subtelomeric positions.

**Fig. 2:**
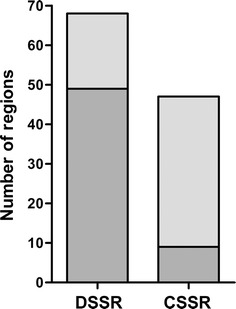
Overlap analysis of high RIIC in *T. cruzi* SSRs. Strand switch regions were identified and their RIIC scores calculated. The bar plot shows the number of DSSR and CSSR regions overlapping high RIIC scoring regions (dark grey) and non-overlapping (light grey)

In order to explore the correlations of high RIIC with SSRs in *T. cruzi*, a necessary first step consisted of identifying SSRs in the *T. cruzi* genome. We restricted the definition of SSRs to those separating polycistronic units containing at least six CDSs and excluded SSRs containing sequencing gaps. Using these stringent criteria, we identified 115 SSRs. Following a scan of the *T. cruzi* genome for high RIIC regions, we computed their overlap with the two types of SSRs (Fig. 2). An association of high RIIC scoring regions and DSSRs was found (72% of the 68 considered DSSRs) being this association specific (Fisher’s test P < 0.0001, Matthews’ correlation coefficient of 0.33). In contrast, only 19% of CSSRs (9 out of 47) were associated with high RIIC scoring regions (not significant Fisher’s test, Matthews’ correlation coefficient of 0.005).

**Fig. 3:**
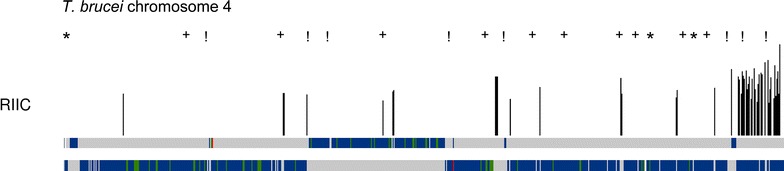
Graphical representation of transcription markers’ signals and sequence dependent curvature RIIC score in *T. brucei.* For *T. brucei* chromosome 4, the graphical representation of regions with RIIC value greater than the selected cutoff (lower panel) are shown above the schematic representation of both chromosome DNA strands depicted in grey, overlaid with CDS features shown in blue. Modified histone locations (H4K10ac) from [17] and base J from [12] are indicated as following: regions associated to H4K10ac but not associated with base J (*); regions associated to H4K10ac and also with base J (!); regions associated with base J and not with H4K10ac (+). Features labeled as ncRNA, snRNA or snoRNAs are shown in green. tRNAs are shown in red. Assembly gaps are shown in brown

The availability of genome-wide epigenetic data associated with transcription starting sites such as modified histones [17], enabled us to directly perform a correlation analysis of genome wide putative TSSs with high RIIC in *T. brucei*, instead to correlate just with DSSRs. So, the number of regions associated with the H4K10ac marker and the extent of their overlap with regions of high RIIC were analyzed (Additional file 1: Table S1 and Additional file 5: Figure S3). Only 34.4% (43 out of 125) of the regions associated with peaks of H4K10ac overlapped with high RIIC. In addition, the univocal association of high RIIC to peaks of H4K10ac is questioned by the great abundance of high RIIC regions not associated with this marker (73.3%, 118 out of 161). This yields a non-significant association for most chromosomes (Fisher test p < 0. 1) and in addition, the global Matthews’ correlation coefficient in this case is only 0.16, while in *Leishmania* it reaches 0.78 [27]. Since a role for the base J in global transcription by RNA polymerase II as well as at telomeric expression sites involved in antigenic variation has been proposed in *T. brucei* [12, 31], we compared its location with the high RIIC regions’ profile (Additional file 1: Table S1 and Additional file 5: Figure S3). Figure 3 shows a representative profile. Globally, a statistically significant overlap of core genome base J containing regions (47.9%, 101 out of 211) with regions of high RIIC was observed and only few of the regions with high RIIC were not associated with base J (37.3%, 60 out of 161) (Fisher’s test P < 0.0001 and Matthews’ correlation coefficient of 0.42).

**Fig. 4:**
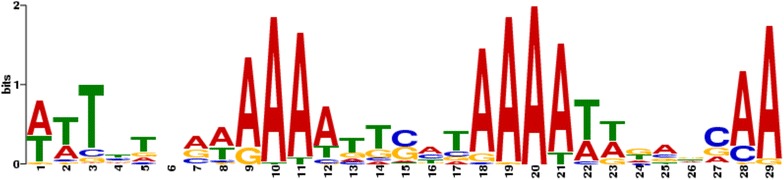
Logo representation of the main motif found by MEME analysis around high curvature peaks in *T. brucei* subtelomeric VSG clusters. The sequences surrounding high IC peaks were analyzed by MEME as described in Materials and “Methods”, and the logo representation for the most significant motif is shown

The striking concentration of high IC at subtelomeric regions prompted us to look at these regions in detail. These regions which inactive VSG genes and pseudogenes, are characterized by a high content of repetitive sequences [32]. Manual inspection of these high IC sequences revealed that they do not correspond to direct or inverted repetitive DNA sequences. This result prompted us to investigate the presence of a sequence motif in the VSG region that would explain the high concentration of sequence-dependent curvature. A pattern of two runs of 4–6 adenine tracts separated by 10 bp constituted the highest scoring motif detected (MEME e-value 4e − 66) (Fig. 4). The motif can be considered a common characteristic in subtelomeric regions since this conserved sequence pattern is present in 50 out of the 103 sequences used for input. However, we found that in the core genome, the ubiquitous short runs of adenines are not clearly associated with high IC peaks (data not shown).

## Discussion

Here we present data demonstrating that regions of high curvature are not randomly distributed along *T. brucei* and *T. cruzi* chromosomes. In both cases the high curvature signals seem more widespread than the profile observed for *Leish*, particularly for *T. brucei*. It is worth noting that the *Leishmania* intrinsic curvature distribution, compared to other organisms, ranging from prokaryotes to humans, including trypanosomes, showed fewer regions with high curvature and a higher density of regions with lower curvature. So the *T. cruzi* and *T. brucei* profile could be attributed to the presence of more regions of high curvature leading to higher median IC (3.32° and 3.52° per helical turn, respectively) compared to *Leishmania* (2.63 per helical turn).

Considering the additive contribution to the DNA curvature of each sequential nucleotides in a given region, we compiled the data by using the region integrated intrinsic curvature score, we have previously developed. The enrichment of high RIIC in DSSRs in *T. cruzi* is suggestive of a putative association of DNA intrinsic curvature with transcription initiation as previously observed in *Leishmania*. Such curvatures may either facilitate the binding of the RNA polymerase and/or help enable the formation of the open DNA complex. Although an enrichment of histone acetylation in the DSSR has been described in *T. cruzi* [16], no studies have been carried out to investigate the presence of TSS markers inside the polycistronic units which prevents a genome wide association study. Considering that high curvature regions are also associated with internal TSSs in *Leishmania*, one can only speculate that a high percentage of the 172 internal high RIIC scoring regions found in *T. cruzi* may also serve as internal TSSs. If this consideration holds true, and some of the 172 internal sites are actual TSS regions, then the number of true positives would increase while the number of false positives will decrease resulting in a more significant association (with an overall increase in the Mathews correlation coefficient).

A difference between the pattern observed here in *T. brucei* and the one previously seen in *Leishmania* is evident. In the latter, the association of high RIIC with transcription initiation is clear [27]. Besides, for this parasite, the location of base J in the core genome corresponds to transcription termination sites [33–35]. In contrast, in *T. brucei*, a high of RIIC is seen with base J, which in this organism is considered a marker of transcription boundaries [12], while the correlation with H4K10ac (marker of transcription initiation) is not significant. Indeed, the coincidence in some regions of high RIIC and H4K10ac enriched regions, might be just the consequence of high association of base J with these regions [12] (68.8%, 86 out of 125). However, since base J is not present in the procyclic stage of the parasite [36], these coincidences may be only restricted to the bloodstream stage. Recently, an attenuation role in gene expression at specific sites within the polycistronic gene units has been proposed for base J in *T. brucei* [34]. It is tempting to speculate that the bulky glucosyl moiety of the base J affects the intrinsic curvature and bendability of regulatory regions involved in facilitating the transcription initiation or processivity. This modification being stage-specific, the location of stage-specific genes downstream to the regions with base J would be expected. Interestingly, in addition to the dispersed internal location of base J, its presence is especially abundant at telomeric and subtelomeric regions in *T. brucei* [36] where stage-specific genes are located.

From our analysis, in phase A runs emerge as a common motif in the subtelomeric regions in *T. brucei.* It has been well established that runs of 4 or more adenines with a 10 bp phasing cause DNA bending [37–40]. The molecular structures of these DNA tracts are unusual and vary depending on the genome context [40]. Multiple roles for the A-tract curvature have been proposed [41]. Among those, A tracts have been implicated in organizing DNA architecture, enhancing the recombination process and assisting chromatin structure. It is also worthwhile noting that curved DNA may vary its shape depending on temperature and physiological changes encompassing the VSG expression silencing in the insect form [42] and tight control in the bloodstream stage.

Although drugs that bind DNA through the recognition and distortion of DNA structure are known to act as trypanocides, their use has been mostly discarded due to the collateral effects on the host [43]. Nonetheless, the work presented here, draws attention to the potential relevance of refining the design of chemotherapeutic agents focusing on the high curvature occurring at regions important for regulation of gene expression and in the case of *T. brucei*, for host immune evasion.

## Conclusions

Highly curved DNA has been recognized as a signal in transcriptional processes both in prokaryotes and eukaryotes (reviewed in [26]). Using the regional integrated intrinsic curvature scoring, we have previously shown the association of regions of high intrinsic curvature with those related to the transcription initiation in *Leishmania* [27]. Here we show that in *T. cruzi*, the divergent strand switches, which are the only regions up to now known to be associated to transcription initiation, are also distinctively characterized by high RIIC scores, supporting the extrapolation of the same conclusion. Meanwhile in *T. brucei,* the association of regions of high intrinsic curvature with the locations of base J was found to be significant. Since base J has been involved in transcription initiation and termination, this result may indicate a role for DNA intrinsic curvature in these processes.

These findings point out to the existence of putative conserved process- and species specific-DNA architectural signals in kinetoplastids. Further studies would be necessary to deeply analyze the recognition signal commonalities and differences that may be involved in different steps of the transcriptional mechanisms.

In addition, in *T. brucei*, the remarkable concentration of regions with high intrinsic curvature at the chromosome subtelomeric regions, where the species-specific genes for the gene family of the highly variable surface glycoproteins are located, may suggest a putative involvement of this structural signal either facilitating the recombinational process or mediating the chromatin silencing and/or granting the vast antigenic variability needed for the efficient evasion of the immune host system that *T. brucei* has developed.

Globally, the data here presented, while establishing particularities within kinetoplastids, underscore the relevance of indirect DNA readout in these ancient eukaryotes.

## Methods

### Data source

The genome data for *T. brucei* strain TREU 927 and the *T. cruzi* CL Brener Esmeraldo-like contigs were downloaded from TritrypDB (version 2.1). *T. brucei* chromosome regions were classified as ‘core’ and ‘subtelomeric’ regions as described in [22].

### Genome-wide intrinsic curvature calculation

The bend.it algorithm [29], kindly provided by Dr. S. Pongor for local runs, was used to obtain the IC values (°/helical turn) for each base on the individual chromosomes which were binned into 200 Kbase segments. The default window size (31 bp), bendability values from nucleosome binding and DNaseI parameters were used. In-house scripts either in Python or in R programming languages were developed to filter and/or further analyze results. For visualization, wiggle (WIG) files were generated for each chromosome.

### Genome-wide regional integrated intrinsic curvature calculation

The RIIC score was calculated as the area under the curvature plot using the Riemann sum. To assess if the *T. brucei* subtelomeric and VSG array regions have a significantly higher RIIC score than the rest of the genome, their RIIC score was compared to a density function representing the population of RIIC scores for equal-length regions in the genome as in [27]. A region was considered highly curved if the RIIC score was bigger than the 95% confidence interval for the population. The genome wide search for high RIIC regions was performed as in [27] with minor modifications. Briefly, every chromosome was scanned for 600 bp regions with a RIIC score greater than the 80th percentile value for that chromosome. For the counting of *T. cruzi* SSRs associated with high RIIC, two criteria were established. Namely, a SSR was not considered if it presented internal sequencing gaps and/or was defined by polycistronic units of less than 6 genes.

### Statistical analysis

To test the association between high RIIC containing and H4K10ac containing regions a contingency matrix was built for each chromosome. The matrix was constructed by classifying genomic regions as containing both signals, only one signal or none and counting each instance. The independence of both variables was tested using the Fisher exact test in R using a *p* value of 0.01 to define significance. The statistical correlation of these regions was tested using the Mathews correlation coefficient working on the previously generated contingency matrix.

### Motif search

For the analysis of motif-based sequence associated to the regions of high intrinsic curvature, the MEME suite was used. The search was performed on a fragment of *T. brucei* chromosome 9 spanning bases from 1 to 319,439 (the VSG array region). A total of 103 sequences surrounding 30 bp each high IC peak were selected for motif discovery. The randomly shuffled sequences were also submitted to the MEME suite as control.

## Additional files

**Additional file 1: Table S1.** Association between high RIIC regions and transcription markers per chromosome.

**Additional file 2.** Wiggle format files containing the positions and magnitude of plotted intrinsic curvature and region integrated intrinsic curvature for *T. cruzi* and *T. brucei* chromosomes.

**Additional file 3: Figure S3.** Graphical representation of sequence dependent curvature in *T. cruzi* chromosomes. The chromosome number is depicted at the top of each page. Upper panel: Bar plots of chromosome positions with an IC value greater than 13 degrees per helical turn. Middle panel: Bar plots of chromosome positions with an RIIC value greater than the selected cutoff. Lower panel: both chromosome DNA strands are depicted in grey, overlaid with CDS features shown in blue. Features labeled as ncRNA, snRNA or snoRNAs are shown in green. tRNAs are shown in red. Assembly gaps are shown in brown.

**Additional file 4: Figure S2.** Graphical representation of sequence dependent curvature in *T. brucei* chromosomes. The chromosome number is depicted at the top of each page. Upper panel: Bar plots of chromosome positions with an IC value greater than 13 degrees per helical turn. Middle panel: Bar plots of chromosome positions with an RIIC value greater than the selected cutoff. Lower panel: both chromosome DNA strands are depicted in grey, overlaid with CDS features shown in blue. Features labeled as ncRNA, snRNA or snoRNAs are shown in green. tRNAs are shown in red. Assembly gaps are shown in brown. Subtelomeric VSG clusters are underlined.

**Additional file 5: Figure S3.** Graphical representation of transcription markers’ signals and sequence dependent curvature RIIC score in *T. brucei.* For each *T. brucei* chromosome, the graphical representation of regions with RIIC value greater than the selected cutoff (lower panel) are shown above the schematic representation of both chromosome DNA strands depicted in grey, overlaid with CDS features shown in blue. Modified histone locations (H4K10ac) from [17] and base J from [12] are indicated as following: regions associated to H4K10ac but not associated with base J (*); regions associated to H4K10ac and also with base J (!); regions associated with base J and not with H4K10ac (+). Features labeled as ncRNA, snRNA or snoRNAs are shown in green. tRNAs are shown in red. Assembly gaps are shown in brown.
