# Supplementary material for: Intrinsic DNA curvature in trypanosomes
Source: BMC Res Notes. 2017 Nov 9;10:585. doi: 10.1186/s13104-017-2908-y (PMC5679330; doi:10.1186/s13104-017-2908-y)
Supplement: Supplementary file 1 — Additional file 1: Table S1. Association between high RIIC regions and transcription markers per chromosome. [file 13104_2017_2908_MOESM1_ESM.docx]

**Supplementary Table 1. Association between high RIIC regions and transcription markers per chromosome.**

|  | H4K10ac + | | H4K10ac - | |  |
| --- | --- | --- | --- | --- | --- |
| Chromosome | RIIC + | RIIC - | RIIC + | RIIC - | p-val (Fisher) |
| 1 | 3 | 4 | 5 | 23 | 0,3117 |
| 2 | 1 | 5 | 7 | 27 | 1,0000 |
| 3 | 3 | 7 | 7 | 38 | 0,3654 |
| 4 | 5 | 6 | 7 | 35 | 0,0984 |
| 5 | 3 | 5 | 4 | 42 | 0,0579 |
| 6 | 2 | 4 | 8 | 40 | 0,3062 |
| 7 | 5 | 7 | 16 | 46 | 0,3031 |
| 8 | 4 | 9 | 15 | 55 | 0,4821 |
| 9 | 2 | 10 | 7 | 83 | 0,2855 |
| 10 | 8 | 12 | 24 | 94 | 0,0820 |
| 11 | 7 | 13 | 18 | 128 | 0,0153 |
|  |  |  |  |  |  |
|  |  |  |  |  |  |
|  | Base J + | | Base J - | |  |
| Chromosome | RIIC + | RIIC - | RIIC + | RIIC - | p-val (Fisher) |
| 1 | 8 | 7 | 0 | 20 | 0,0003 |
| 2 | 7 | 7 | 1 | 25 | 0,0012 |
| 3 | 9 | 8 | 1 | 37 | 0,0001 |
| 4 | 11 | 7 | 1 | 34 | 0,0001 |
| 5 | 7 | 12 | 0 | 35 | 0,0003 |
| 6 | 4 | 6 | 6 | 38 | 0,0746 |
| 7 | 14 | 7 | 7 | 46 | 0,0001 |
| 8 | 10 | 14 | 9 | 50 | 0,0188 |
| 9 | 5 | 13 | 4 | 80 | 0,0080 |
| 10 | 13 | 13 | 19 | 93 | 0,0012 |
| 11 | 13 | 16 | 12 | 98 | 0,0001 |

+ and - signs indicate presence or absence respectively. The p-value of a Fisher’s exact test is indicated in the last column. Most chromosomes show significant associations with RIIC in the case of Base J (p < 0.01).
