# Supplementary material for: Intrinsic DNA curvature in trypanosomes
Source: BMC Res Notes. 2017 Nov 9;10:585. doi: 10.1186/s13104-017-2908-y (PMC5679330; doi:10.1186/s13104-017-2908-y)
Supplement: Supplementary file 3 — Additional file 3: Figure S3. Graphical representation of sequence dependent curvature in T. cruzi chromosomes. The chromosome number is depicted at the top of each page. Upper panel: Bar plots of chromosome positions with an IC value greater than 13 degrees per helical turn. Middle panel: Bar plots of chromosome positions with an RIIC value greater than the selected cutoff. Lower panel: both chromosome DNA strands are depicted in grey, overlaid with CDS features shown in blue. Features labeled as ncRNA, snRNA or snoRNAs are shown in green. tRNAs are shown in red. Assembly gaps are shown in brown. [file 13104_2017_2908_MOESM3_ESM.pdf]

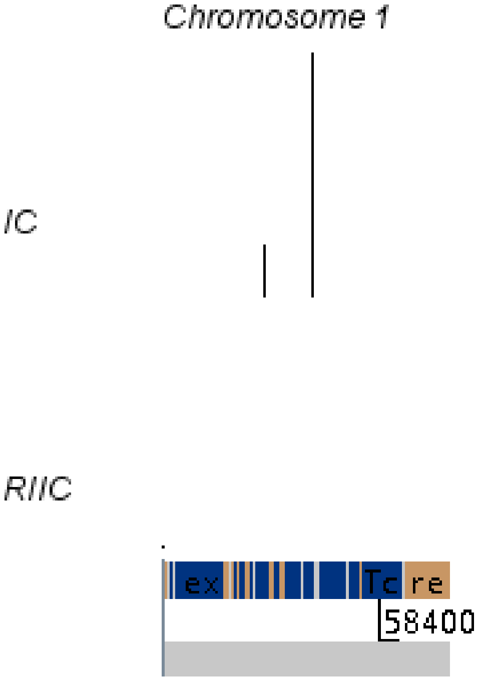
Chromosome 1
IC
RIIC
ex
Tc
re
58400

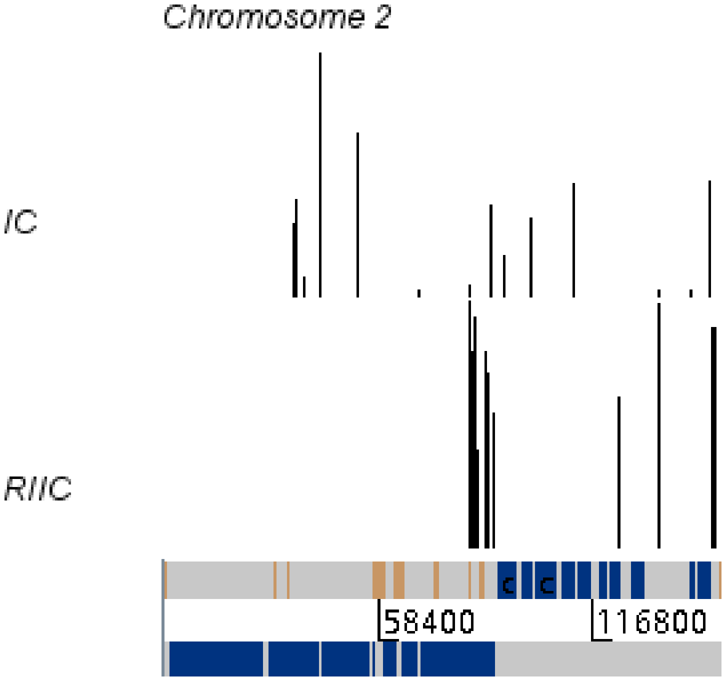

Chromosome 2
IC
RIIC
c
c
58400
116800

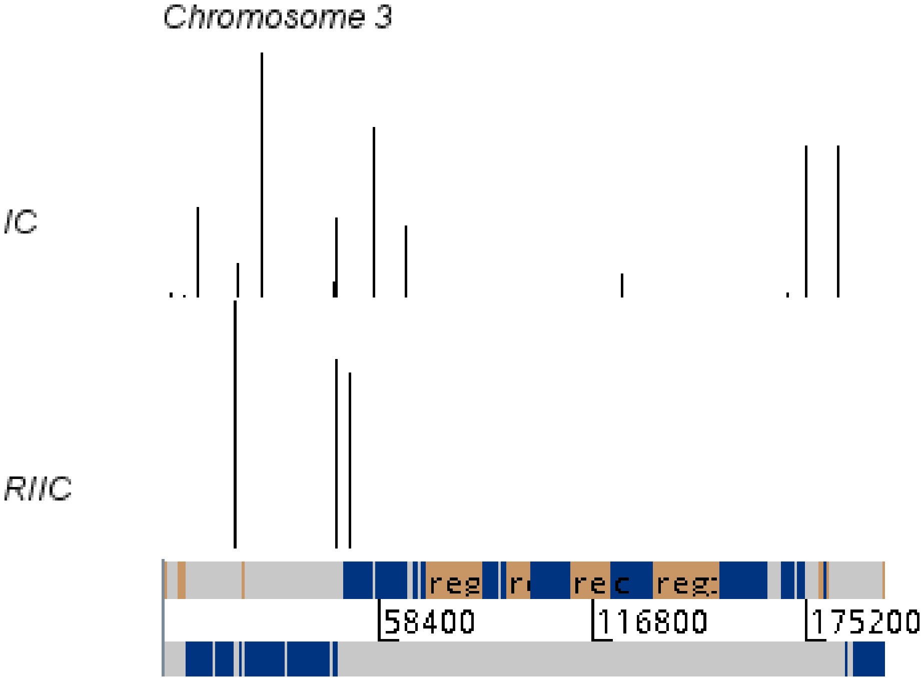

Chromosome 3
IC
RIIC
58400
116800
175200

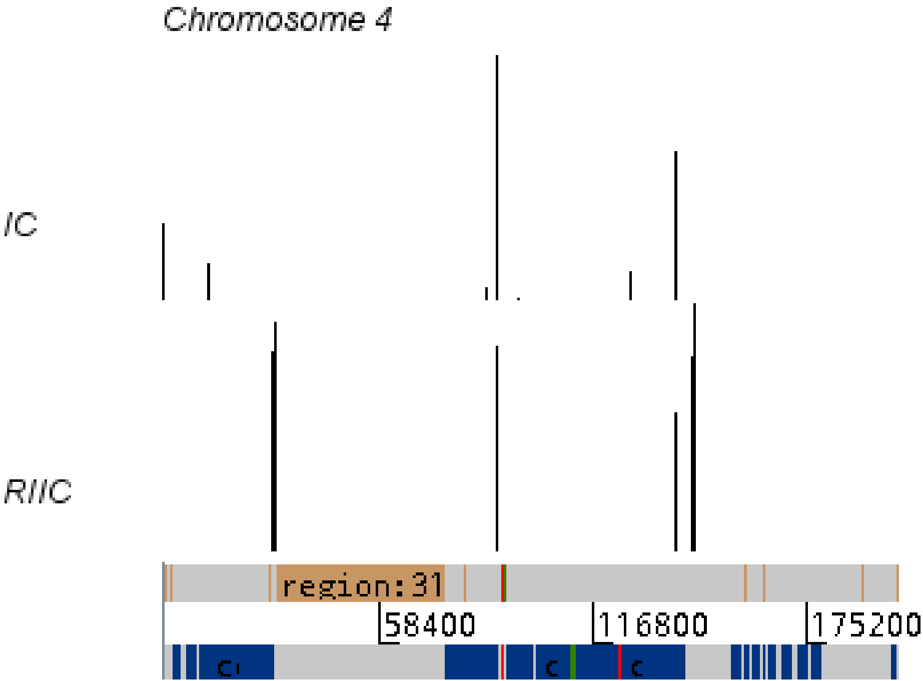
Chromosome 4
IC
RIIC
region:31
58400
116800
175200

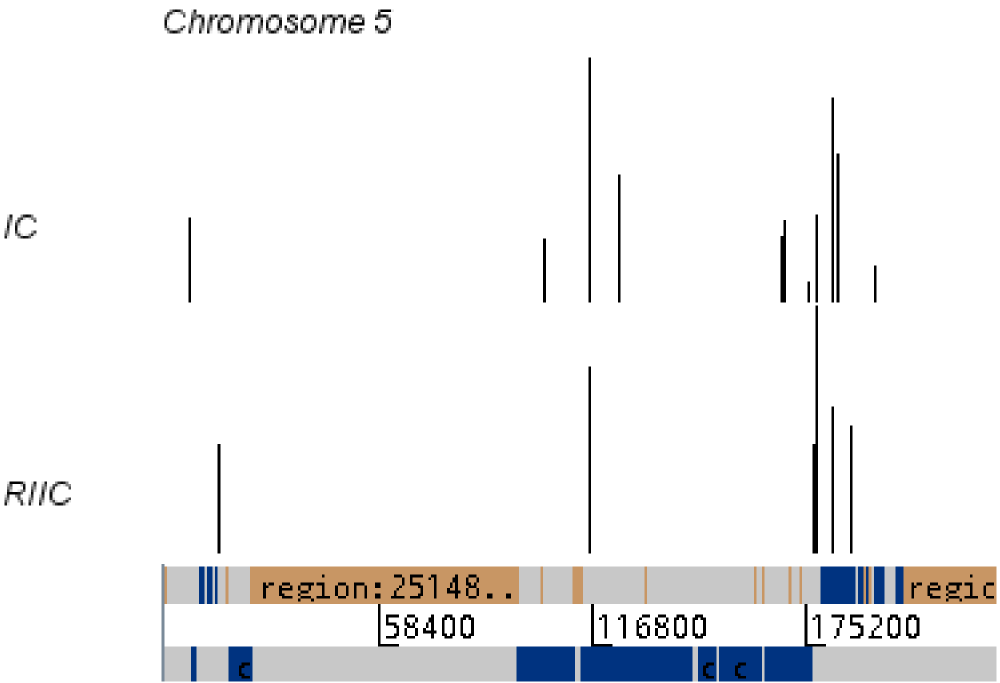

Chromosome 5
IC
RIIC
region:25148..
regic
58400
116800
175200
c
c
c

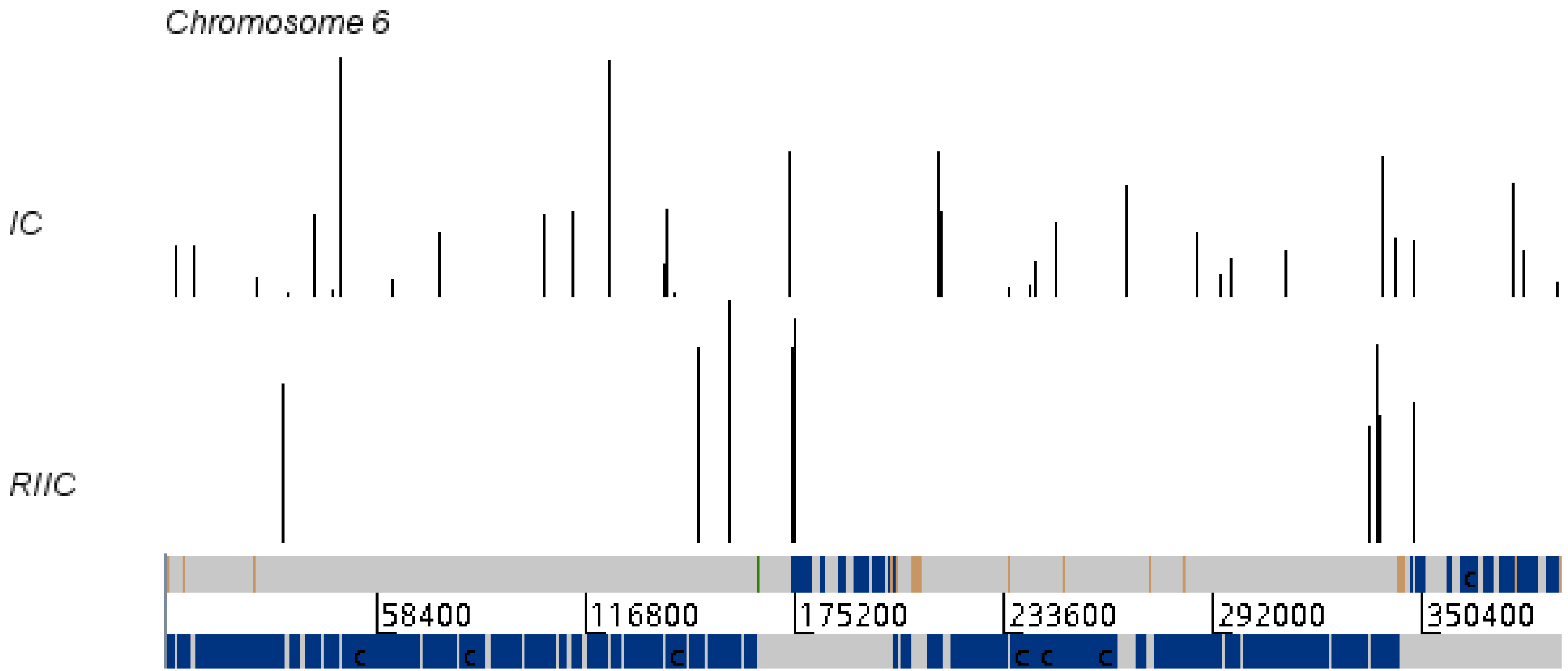
Chromosome 6
IC
RIIC
58400
116800
175200
233600
292000
350400

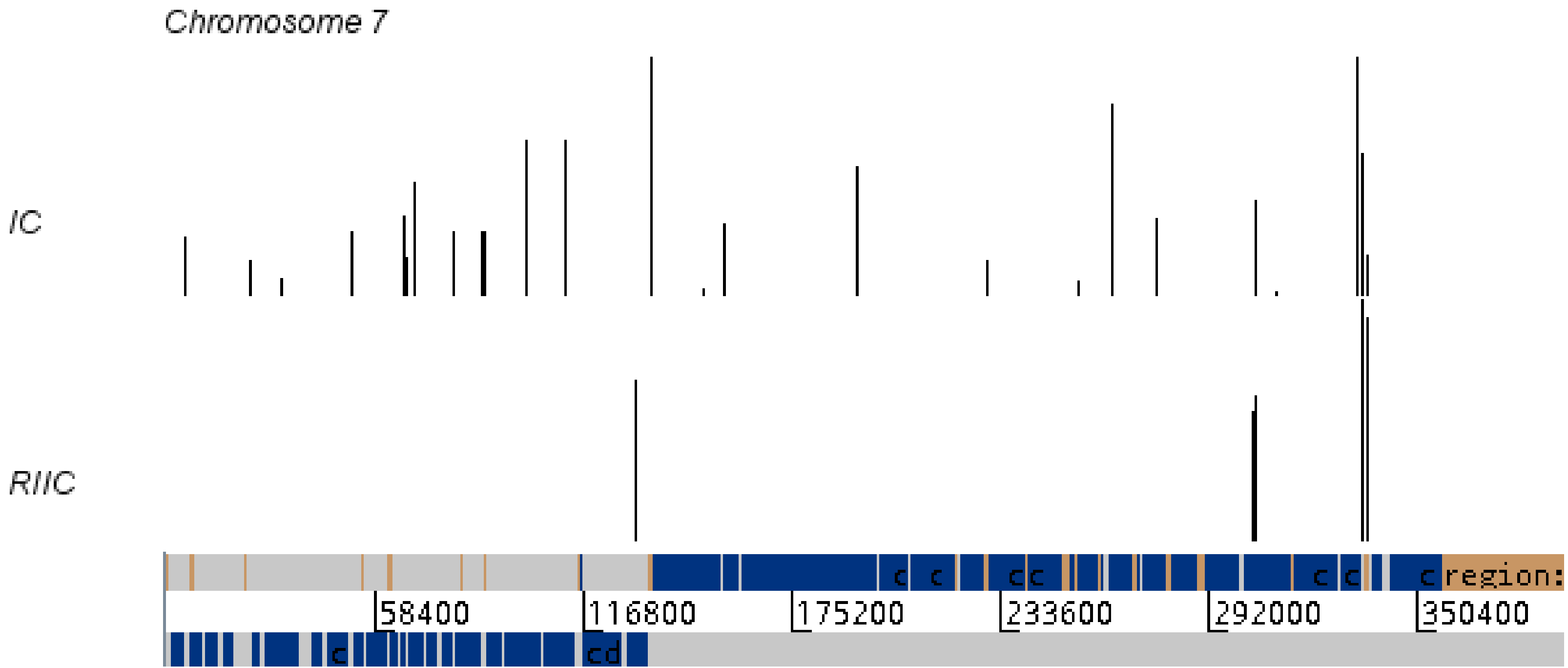
Chromosome 7
IC
RIIC
region:
58400
116800
175200
233600
292000
350400

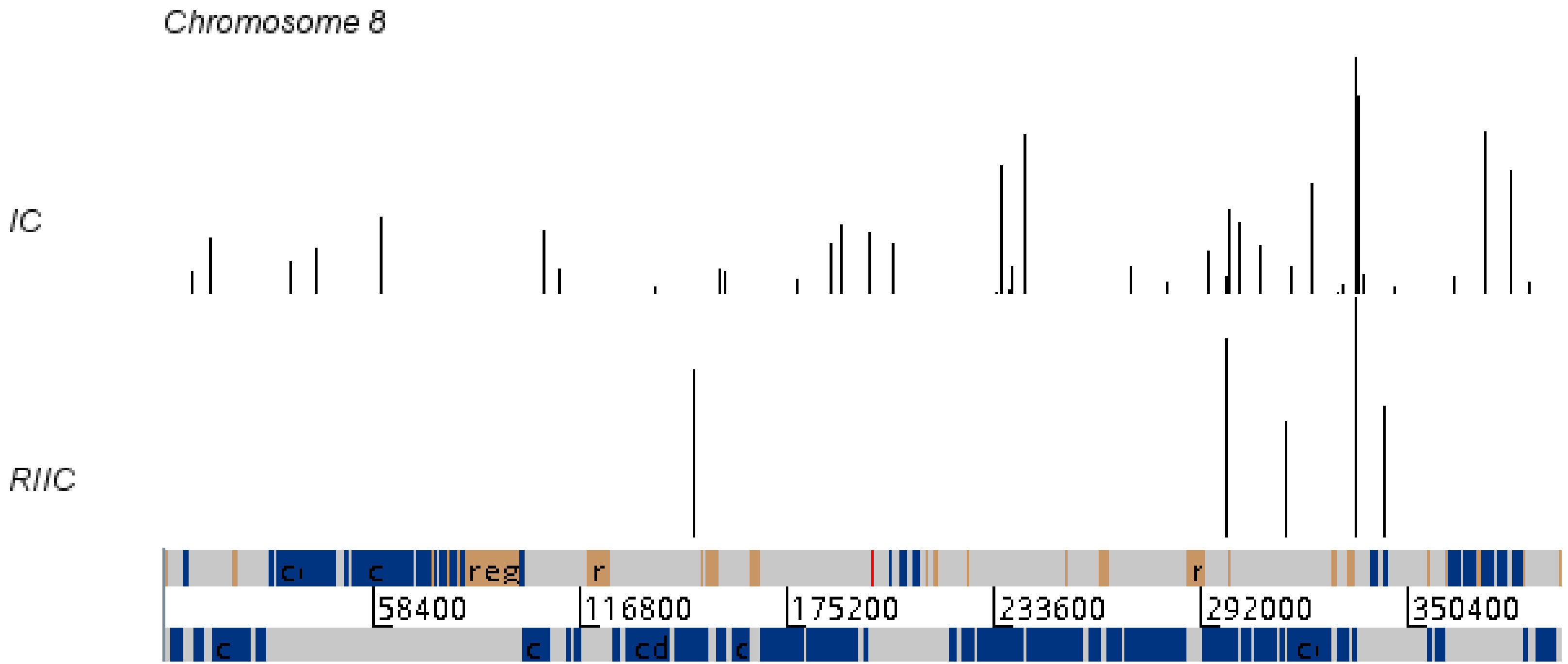

Chromosome 8
IC
RIIC
reg
58400
116800
175200
233600
292000
350400

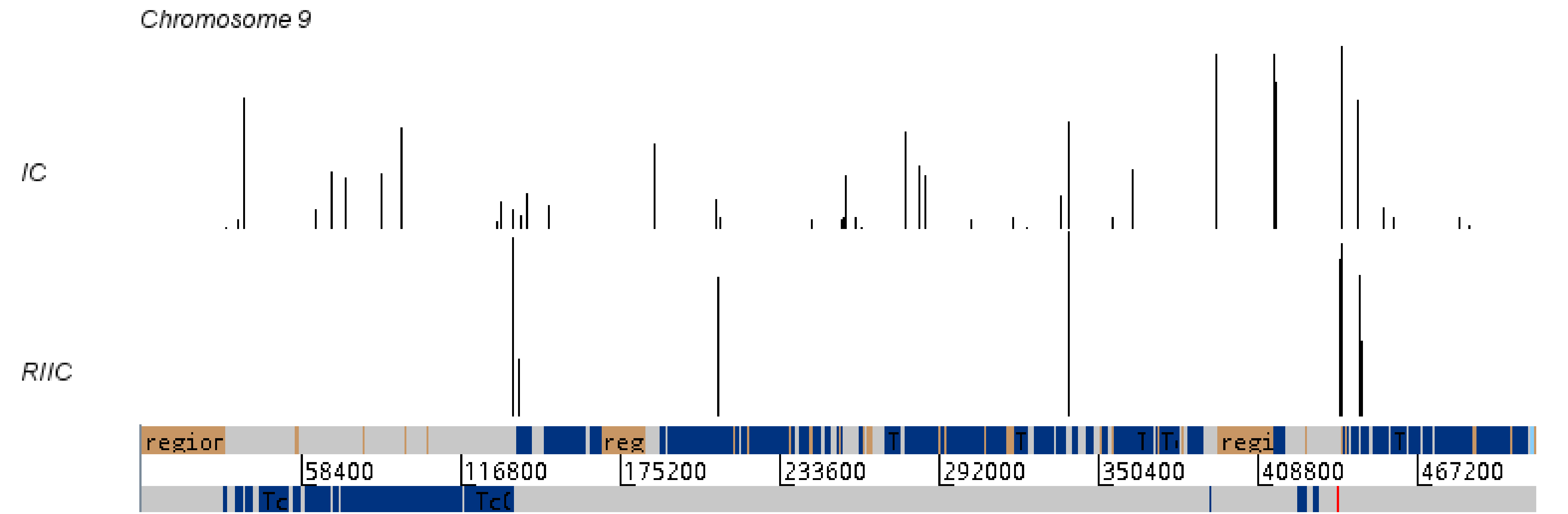
Chromosome 9
IC
RIIC
regior
reg
regi
58400
116800
175200
233600
292000
350400
408800
467200

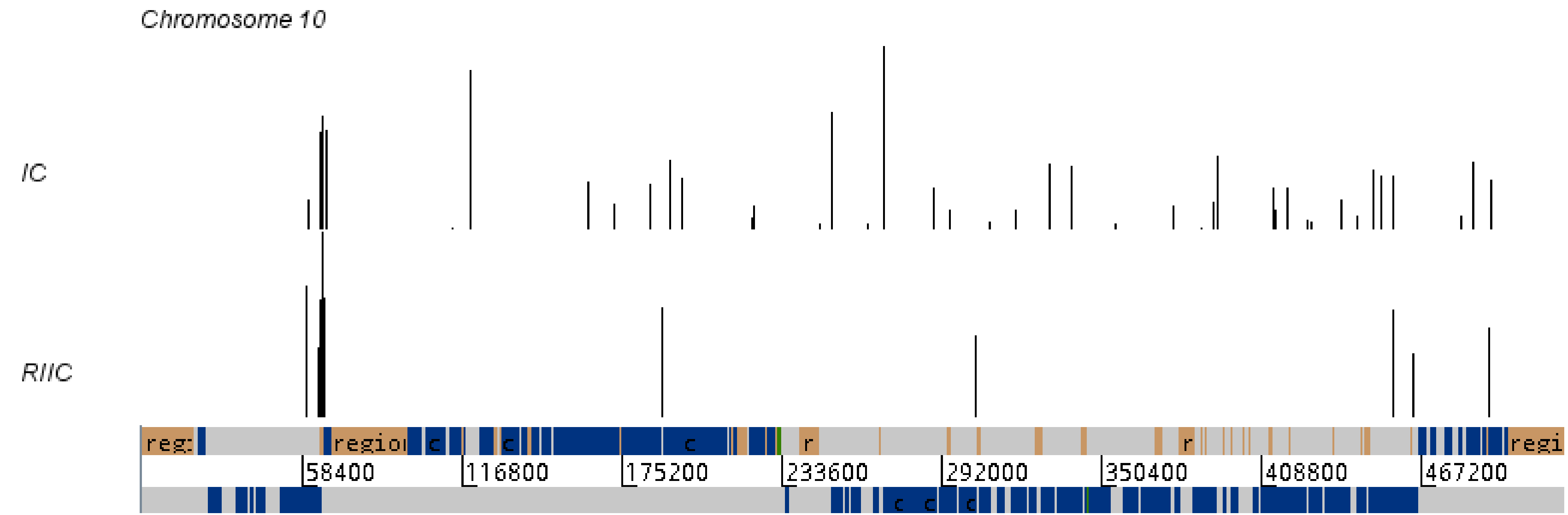
Chromosome 10
IC
RIIC
58400
116800
175200
233600
292000
350400
408800
467200

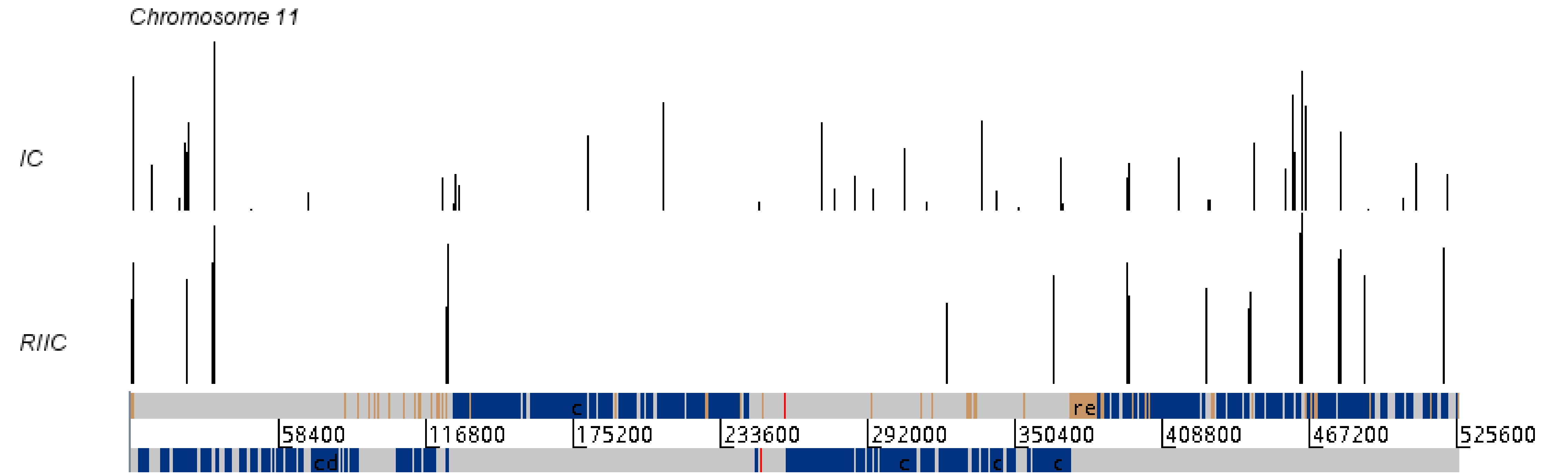
Chromosome 11
IC
RIIC
c
re
58400
116800
175200
233600
292000
350400
408800
467200
525600
cd
c
c
c

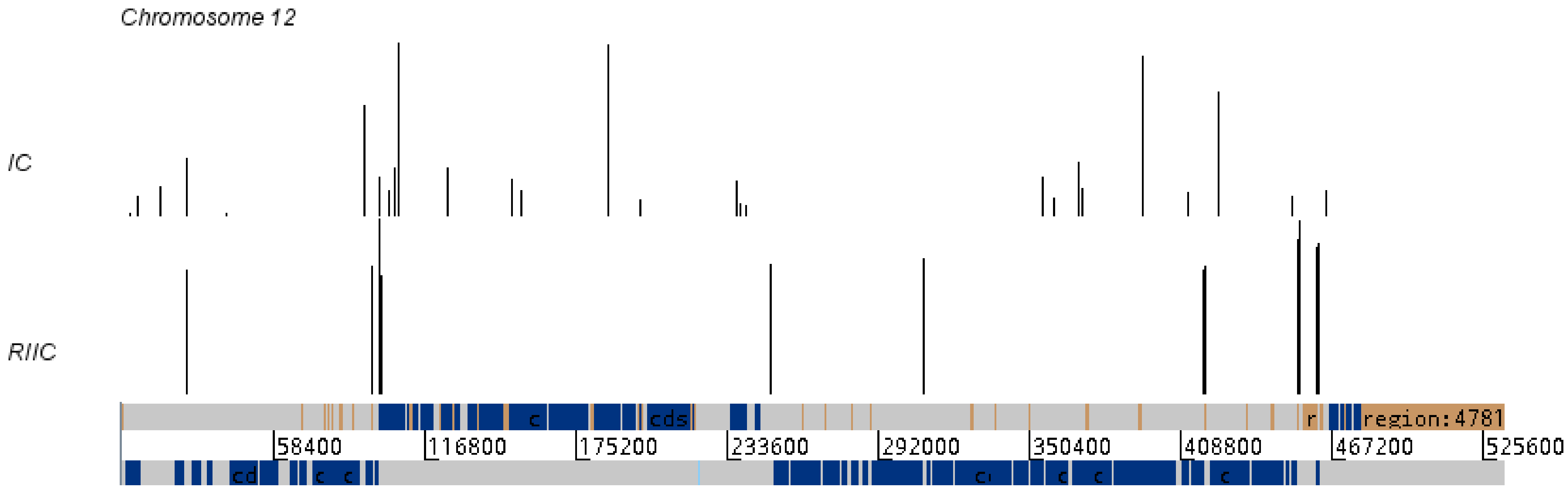
Chromosome 12
IC
RIIC
cds
region:4781
58400
116800
175200
233600
292000
350400
408800
467200
525600

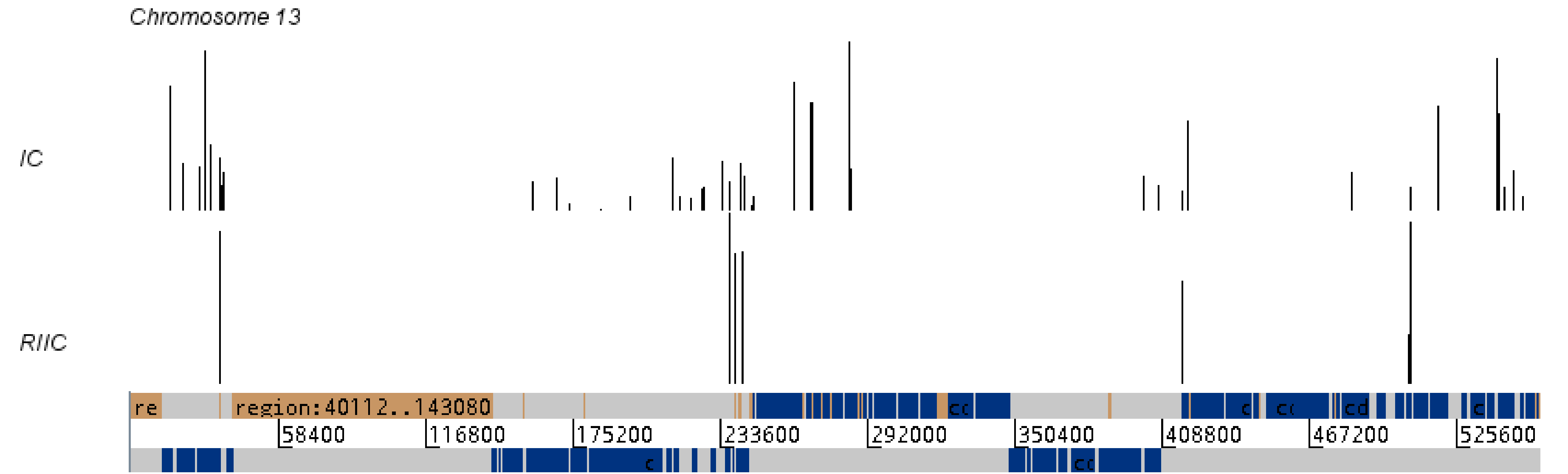
Chromosome 13
IC
RIIC
re
region:40112..143080
cc
c
cc
cd
c
58400
116800
175200
233600
292000
350400
408800
467200
525600
c
cc

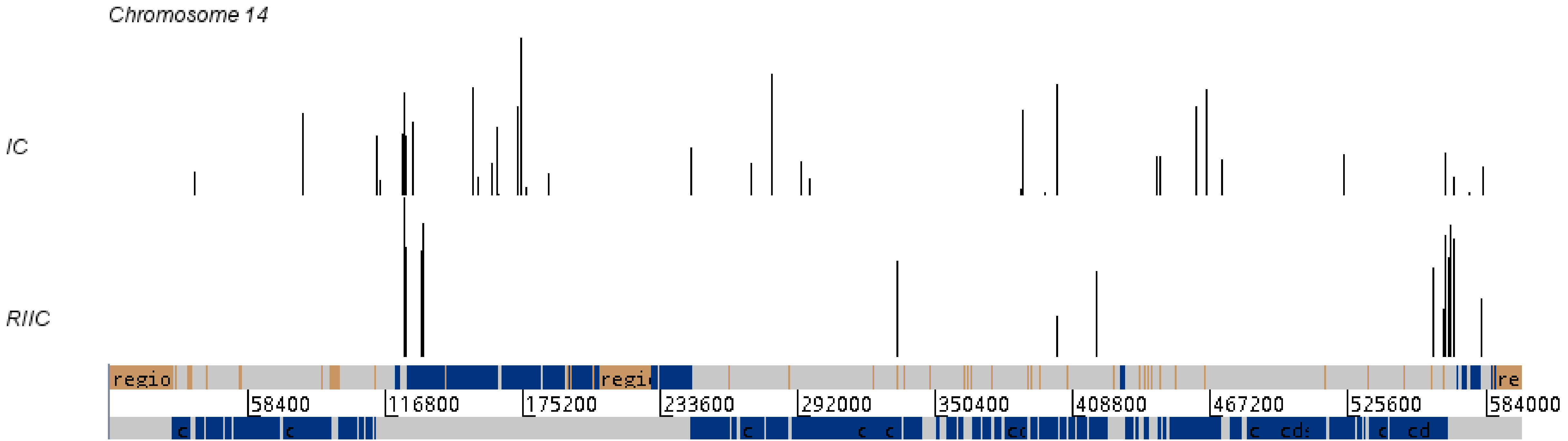
Chromosome 14
IC
RIIC
regio
regi
re
58400
116800
175200
233600
292000
350400
408800
467200
525600
584000

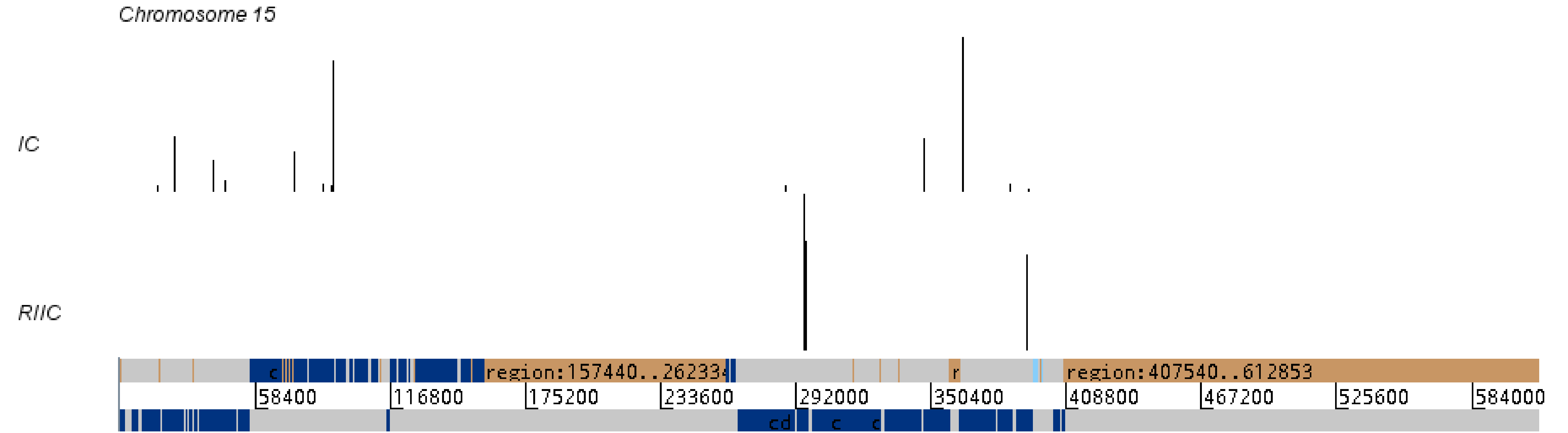
Chromosome 15
IC
RIIC
region:157440..26233
region:407540..612853
58400
116800
175200
233600
292000
350400
408800
467200
525600
584000

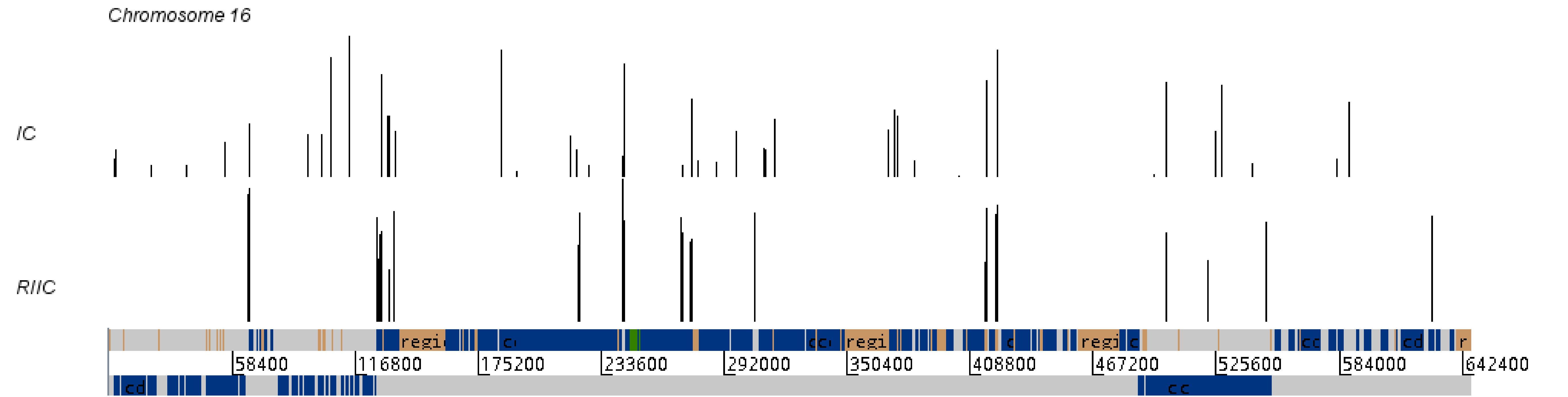
Chromosome 16
IC
RIIC
58400
116800
175200
233600
292000
350400
408800
467200
525600
584000
642400

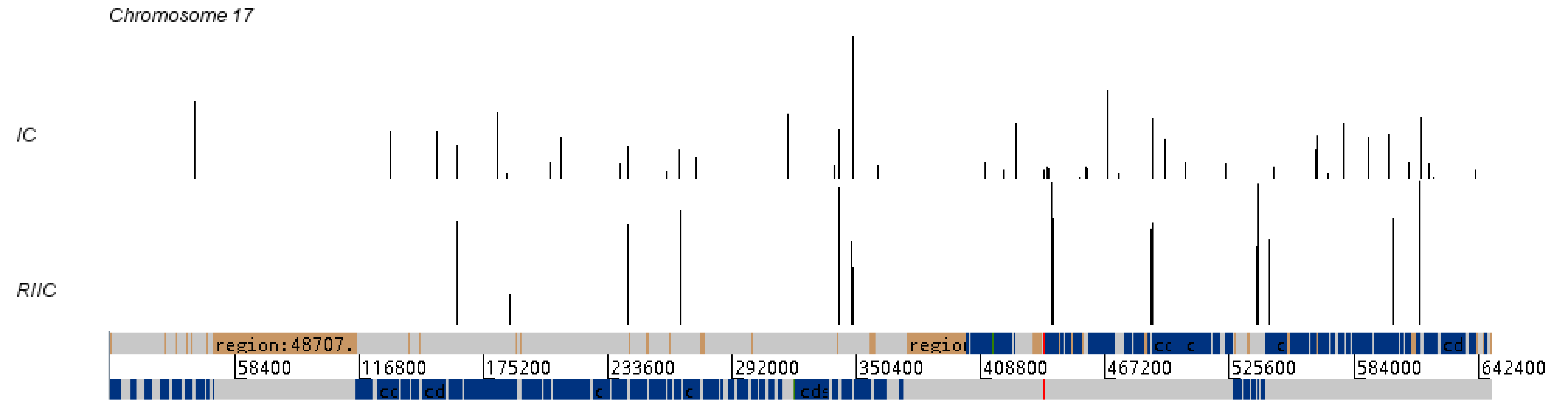

Chromosome 17
IC
RIIC
region:48707.
regio
cc
c
c
cd
58400
116800
175200
233600
292000
350400
408800
467200
525600
584000
642400
cc
cd
c
c
cds

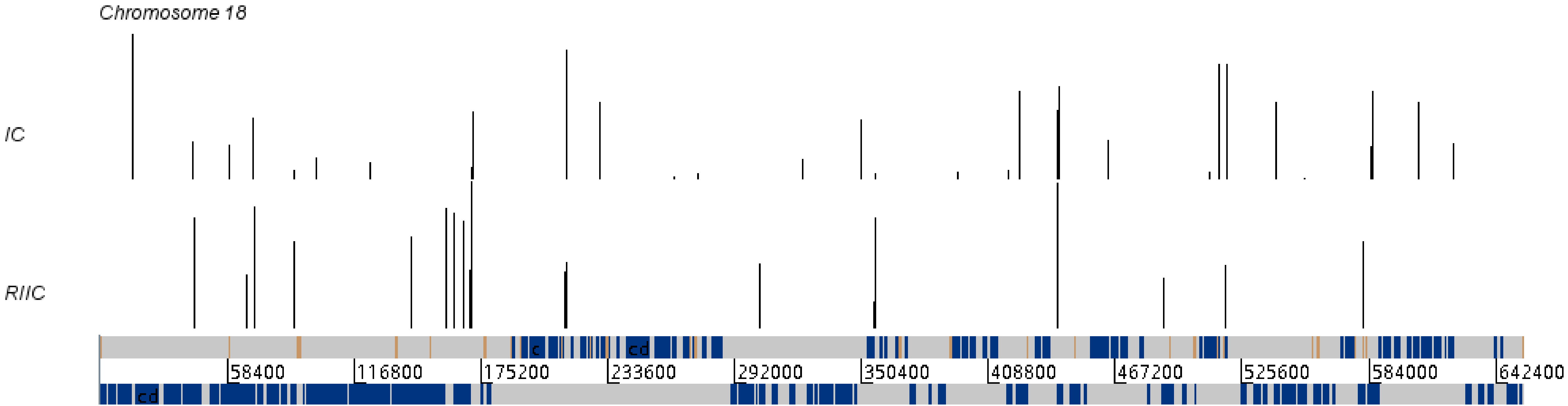

Chromosome 18
IC
RIIC
c
cd
58400
116800
175200
233600
292000
350400
408800
467200
525600
584000
642400
cd

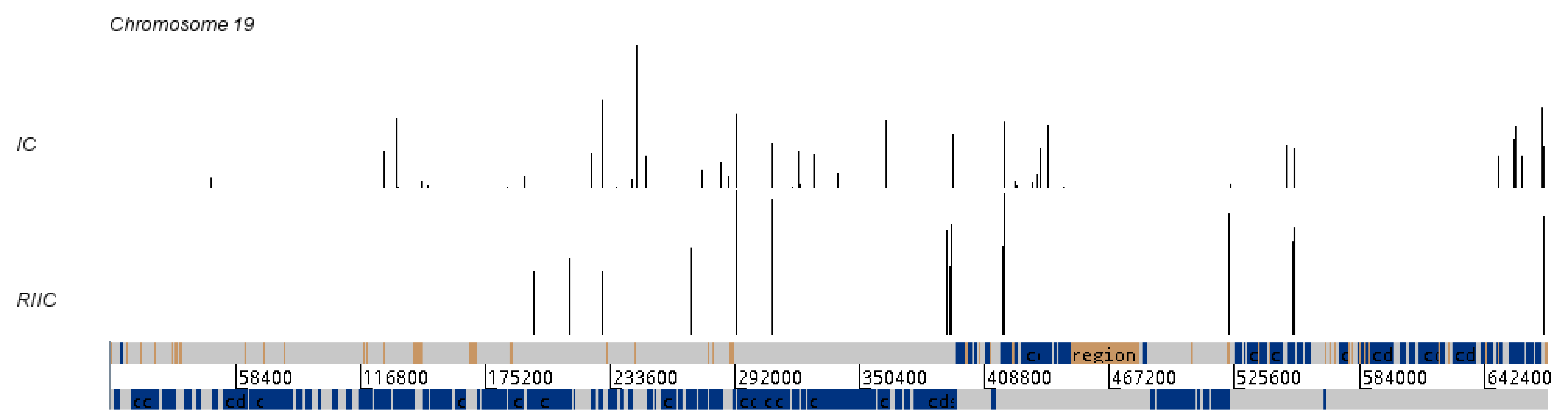

Chromosome 19
IC
RIIC
region
58400
116800
175200
233600
292000
350400
408800
467200
525600
584000
642400

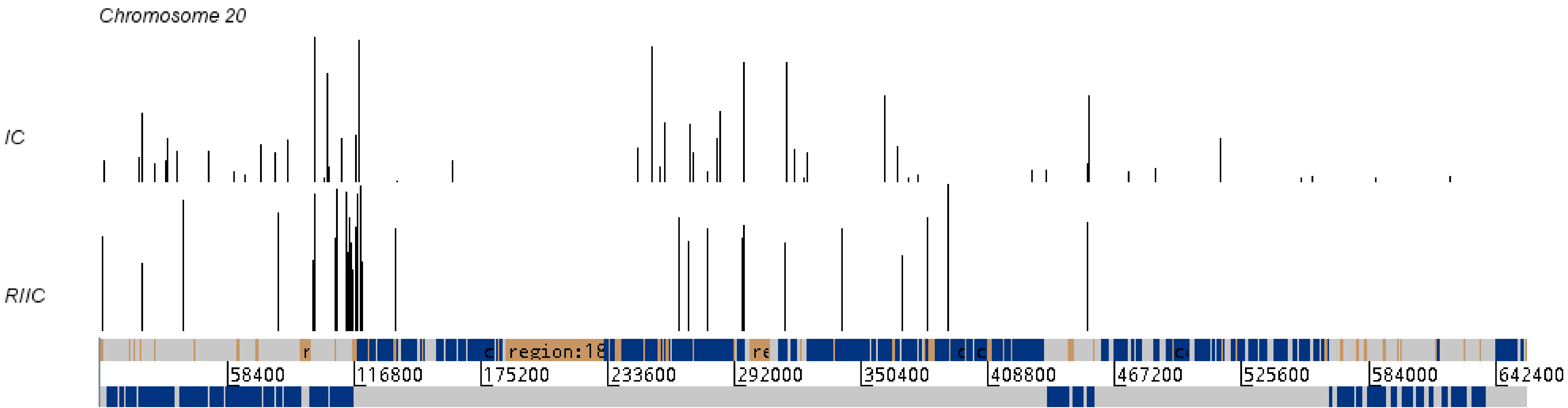

Chromosome 20
IC
RIIC
region:18
58400
116800
175200
233600
292000
350400
408800
467200
525600
584000
642400

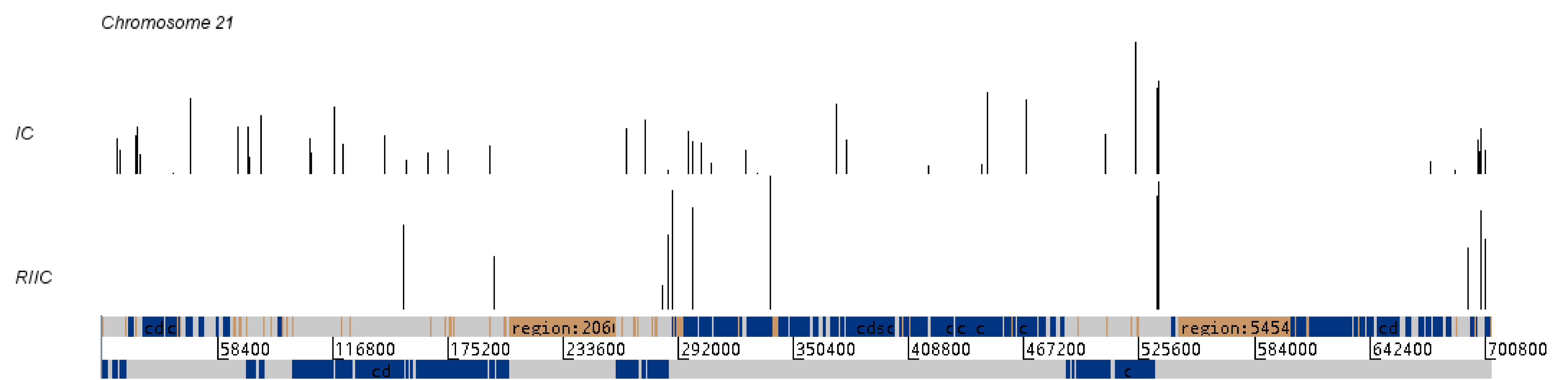
Chromosome 21
IC
RIIC
cdc
region:206
cdsc
c c c
c
region:5454
cd
58400
116800
175200
233600
292000
350400
408800
467200
525600
584000
642400
700800
cd
c

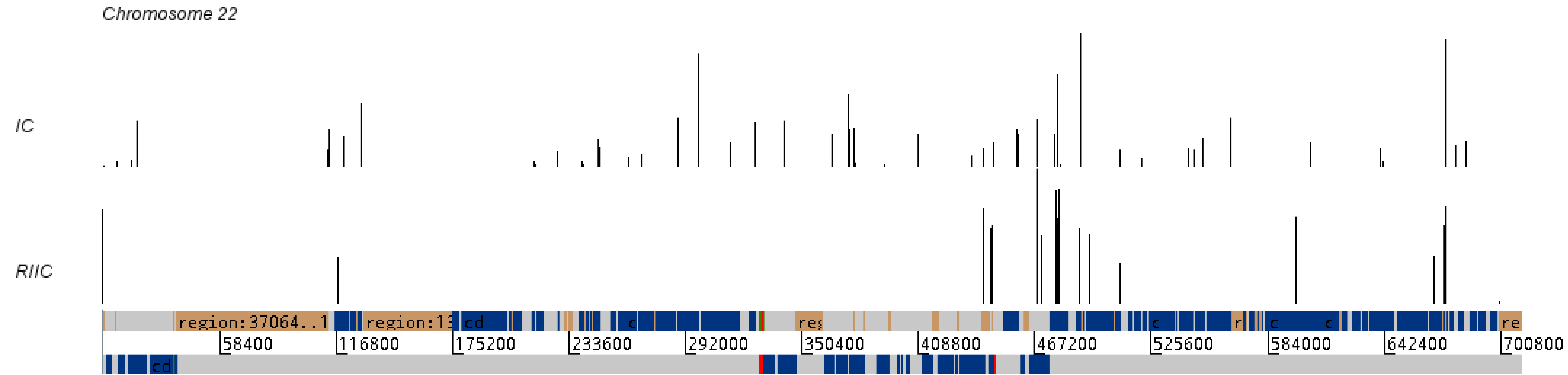
Chromosome 22
IC
RIIC
region:37064..1
region:13
cd
58400
116800
175200
233600
292000
350400
408800
467200
525600
584000
642400
700800
cd

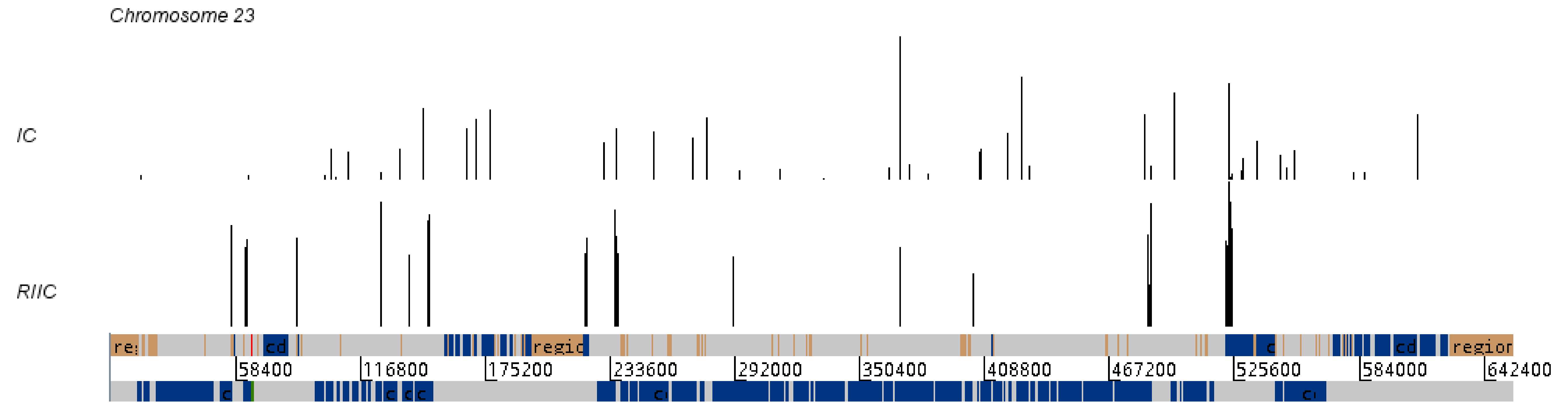

Chromosome 23
IC
RIIC
58400
116800
175200
233600
292000
350400
408800
467200
525600
584000
642400

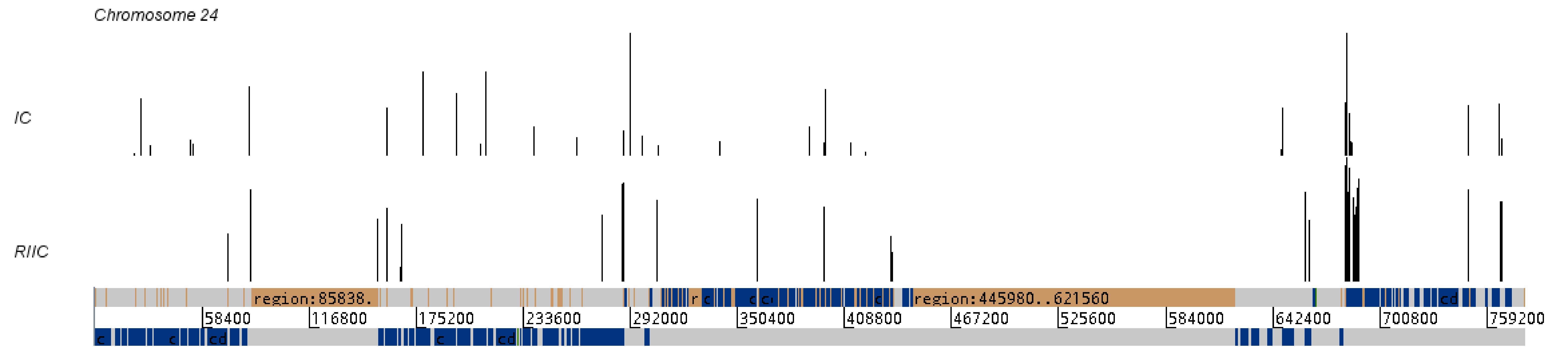

Chromosome 24
IC
RIIC
region:85838.
region:445980..621560
58400
116800
175200
233600
292000
350400
408800
467200
525600
584000
642400
700800
759200

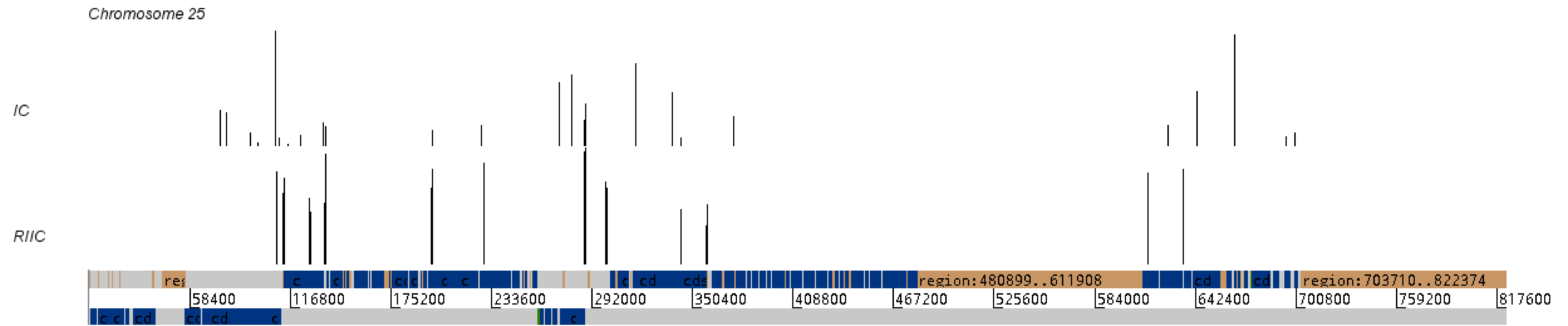
Chromosome 25
IC
RIIC
region:480899..611908
region:703710..822374
58400
116800
175200
233600
292000
350400
408800
467200
525600
584000
642400
700800
759200
817600

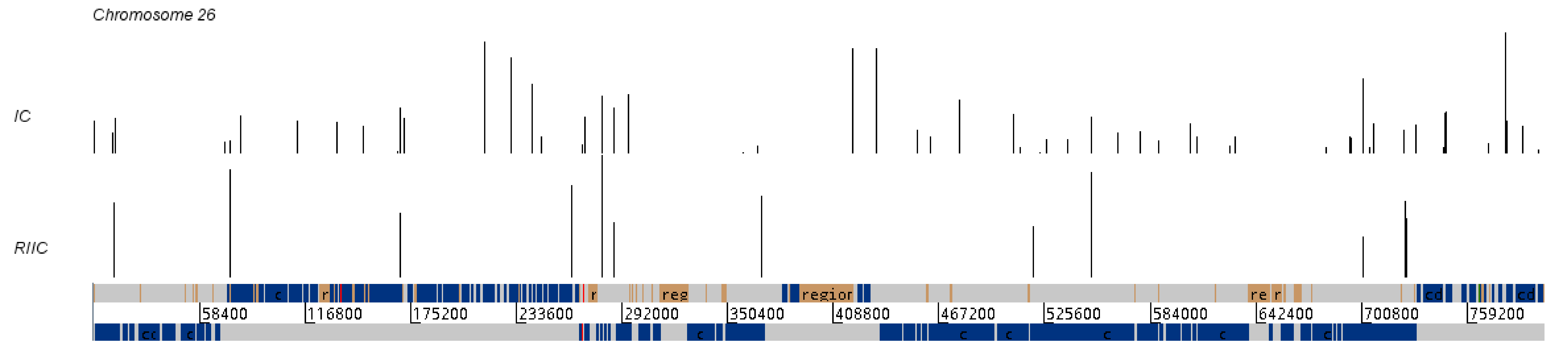
Chromosome 26
IC
RIIC
c
r
r
reg
region
re
r
cd
cd
58400
116800
175200
233600
292000
350400
408800
467200
525600
584000
642400
700800
759200
cc
c
c
c
c
c
c
c

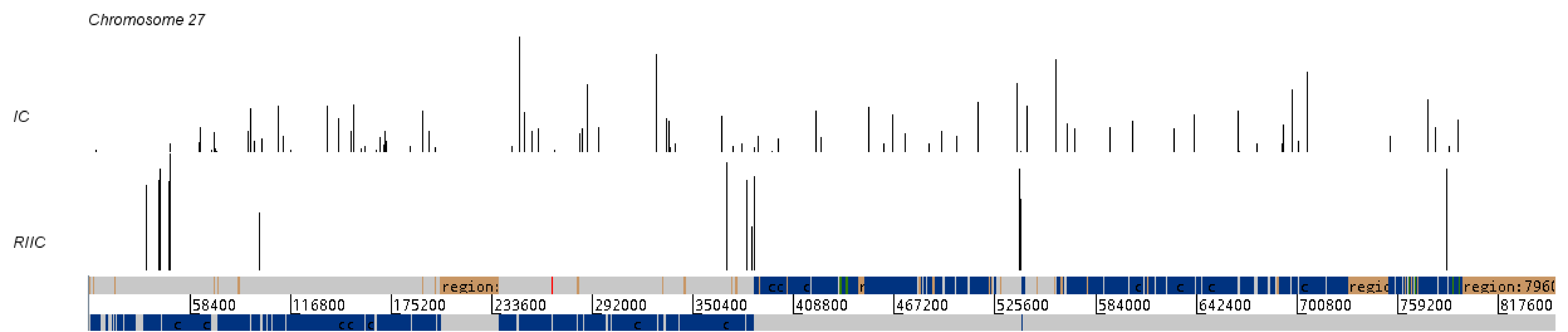
Chromosome 27
IC
RIIC
58400
116800
175200
233600
292000
350400
408800
467200
525600
584000
642400
700800
759200
817600

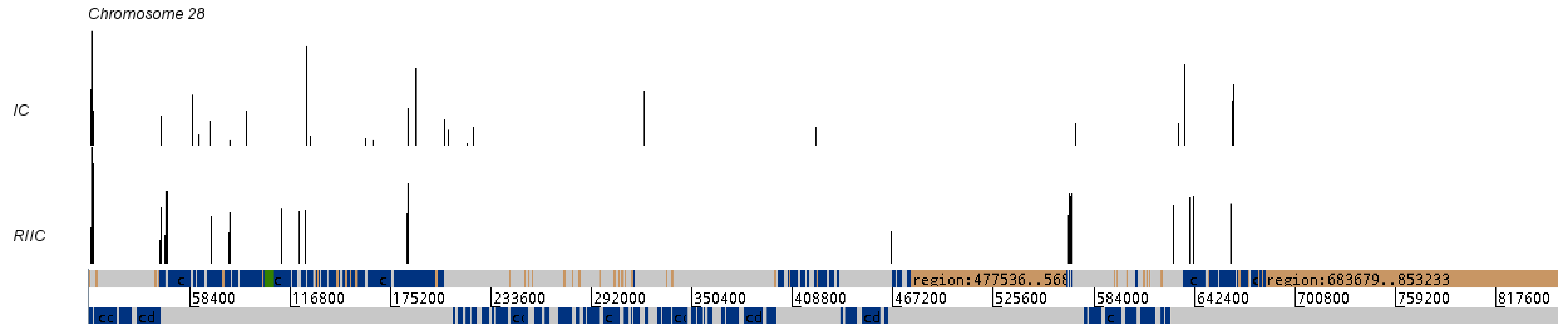
Chromosome 28
IC
RIIC
region:477536..568
region:683679..853233
58400
116800
175200
233600
292000
350400
408800
467200
525600
584000
642400
700800
759200
817600

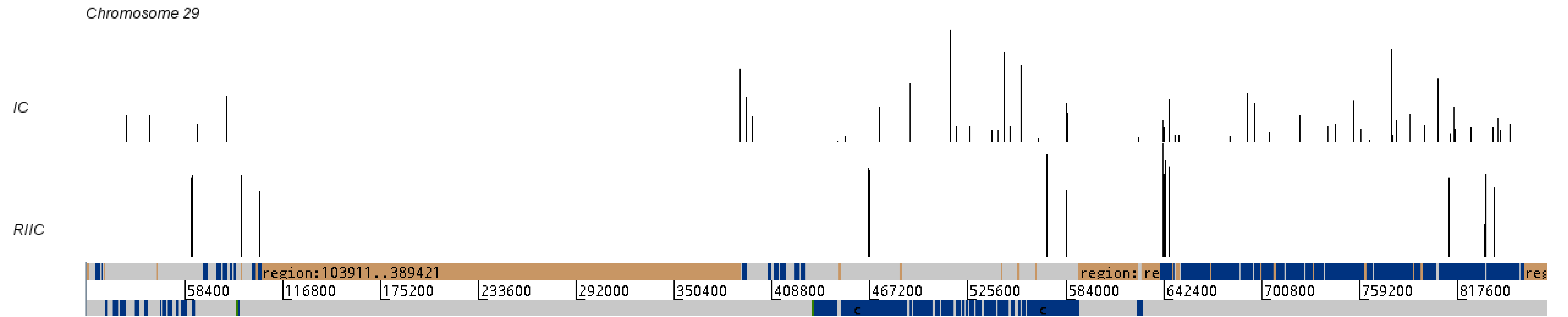
Chromosome 29
IC
RIIC
region:103911..389421
region:
58400
116800
175200
233600
292000
350400
408800
467200
525600
584000
642400
700800
759200
817600

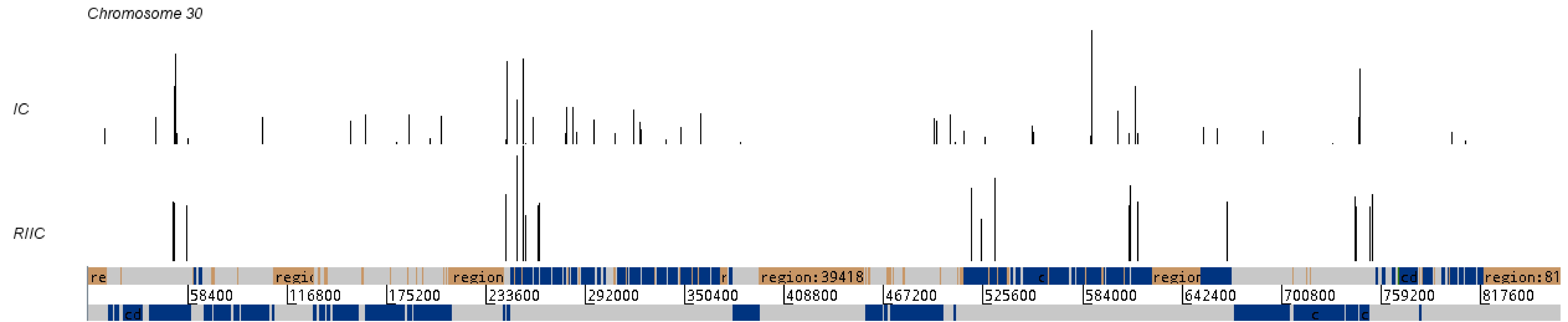

Chromosome 30
IC
RIIC
58400
116800
175200
233600
292000
350400
408800
467200
525600
584000
642400
700800
759200
817600

Chromosome 31

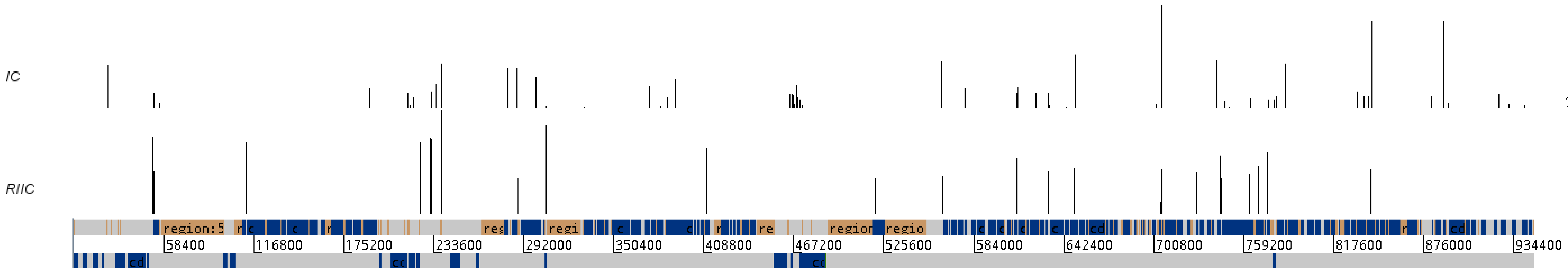

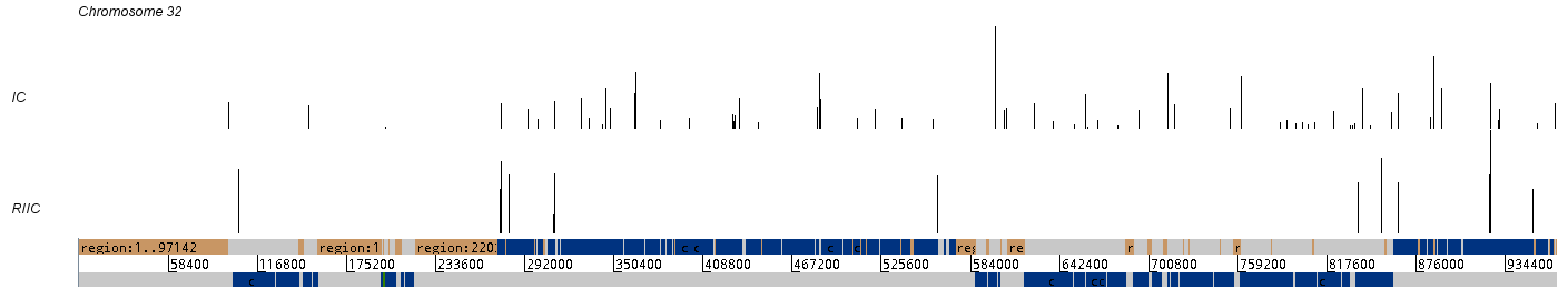
Chromosome 32
IC
RIIC
region:1..97142
58400
116800
175200
233600
292000
350400
408800
467200
525600
584000
642400
700800
759200
817600
876000
934400

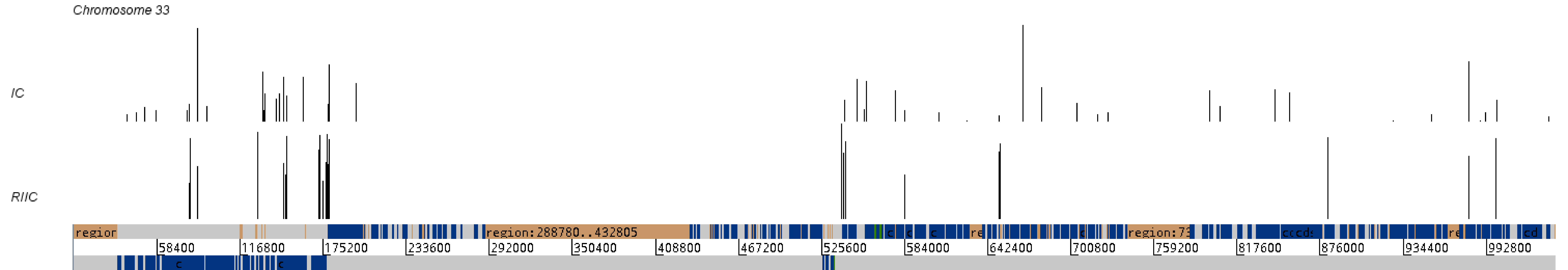
Chromosome 33
IC
RIIC
region:288780..432805
58400
116800
175200
233600
292000
350400
408800
467200
525600
584000
642400
700800
759200
817600
876000
934400
992800

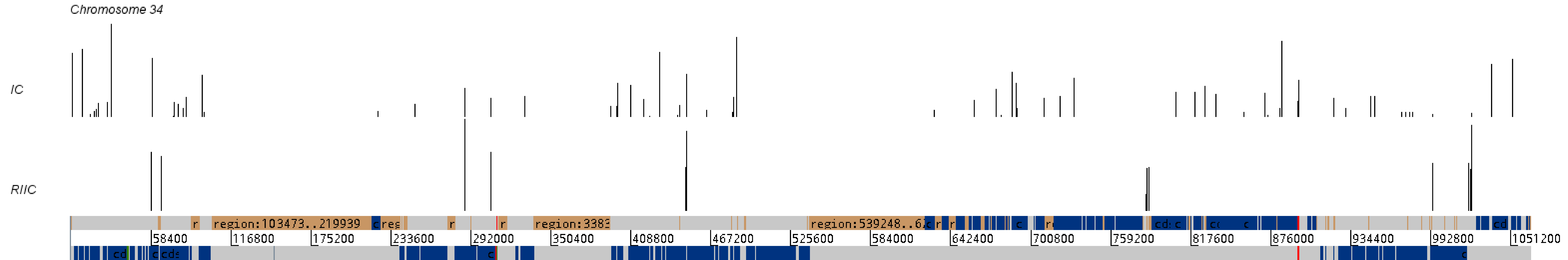
Chromosome 34
IC
RIIC
region:103473..219939
region:3383
region:539248..6
58400
116800
175200
233600
292000
350400
408800
467200
525600
584000
642400
700800
759200
817600
876000
934400
992800
1051200

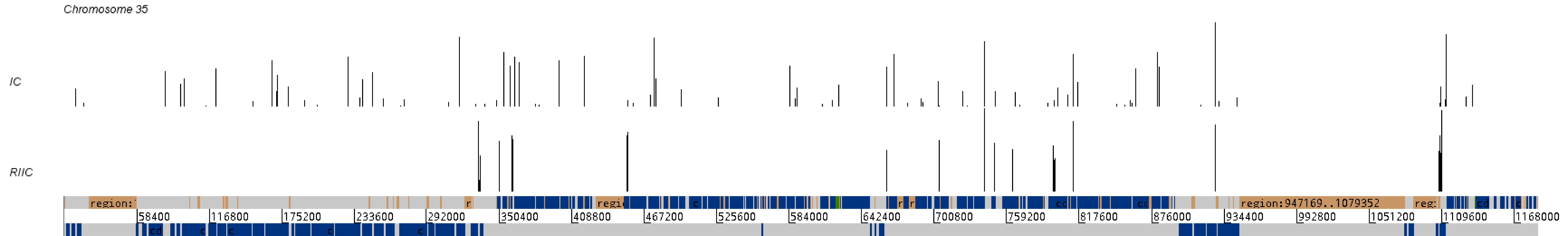
Chromosome 35
IC
RIIC
region:947169..1079352
58400
116800
175200
233600
292000
350400
408800
467200
525600
584000
642400
700800
759200
817600
876000
934400
992800
1051200
1109600
1168000

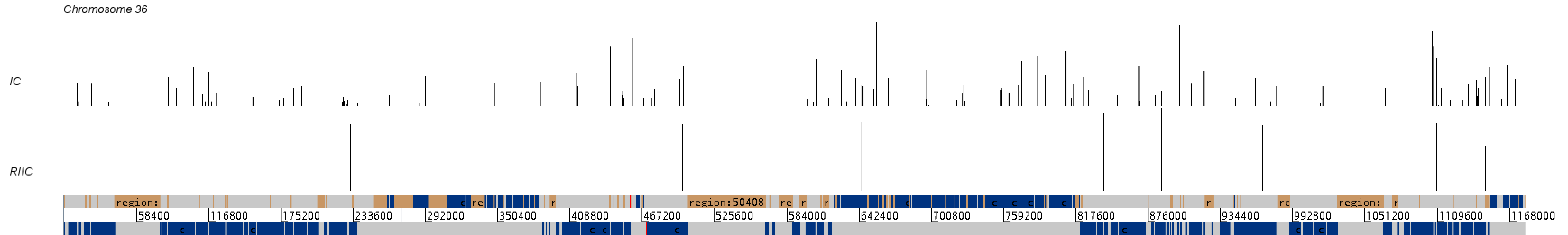
Chromosome 36
IC
RIIC
region:
region:50408
region:
58400
116800
175200
233600
292000
350400
408800
467200
525600
584000
642400
700800
759200
817600
876000
934400
992800
1051200
1109600
1168000

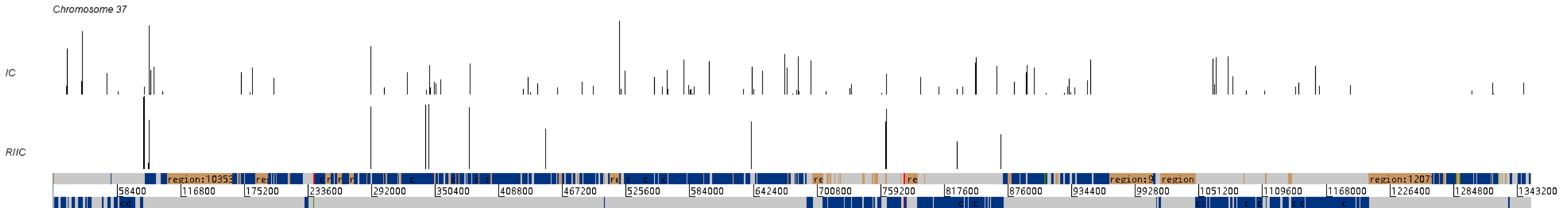
Chromosome 37
IC
RIIC
58400
116800
175200
233600
292000
350400
408800
467200
525600
584000
642400
700800
759200
817600
876000
934400
992800
1051200
1109600
1168000
1226400
1284800
1343200

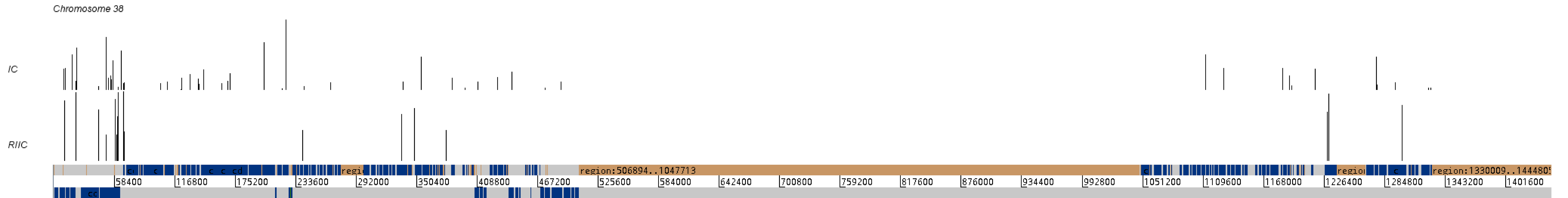

Chromosome 38
IC
RIIC
region:506894..1047713
region:1330009..1444805
58400
116800
175200
233600
292000
350400
408800
467200
525600
584000
642400
700800
759200
817600
876000
934400
992800
1051200
1109600
1168000
1226400
1284800
1343200
1401600

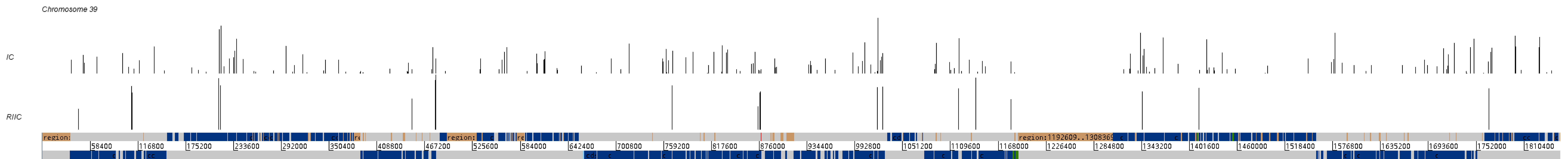
Chromosome 39
IC
RIIC
58400
116800
175200
233600
292000
350400
408800
467200
525600
584000
642400
700800
759200
817600
876000
934400
992800
1051200
1109600
1168000
1226400
1284800
1343200
1401600
1460000
1518400
1576800
1635200
1693600
1752000
1810400

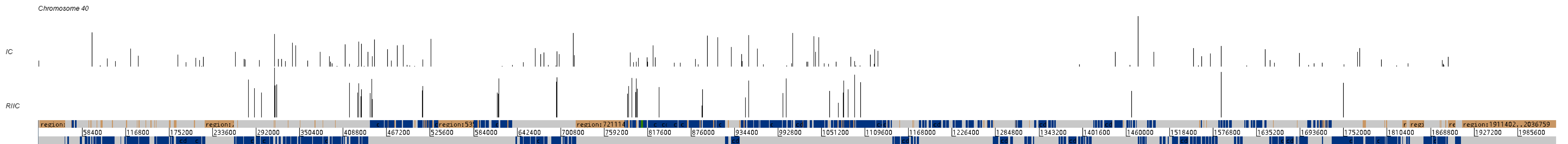
Chromosome 40
IC
RIIC
region:721114
region:1911402..2036759
58400
116800
175200
233600
292000
350400
408800
467200
525600
584000
642400
700800
759200
817600
876000
934400
992800
1051200
1109600
1168000
1226400
1284800
1343200
1401600
1460000
1518400
1576800
1635200
1693600
1752000
1810400
1868800
1927200
1985600

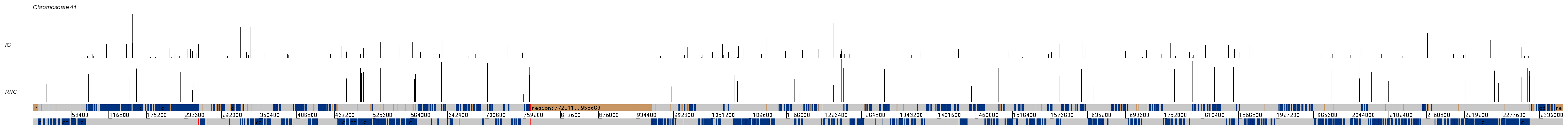
Chromosome 41
IC
RIIC
region:772211..958683
58400
116800
175200
233600
292000
350400
408800
467200
525600
584000
642400
700800
759200
817600
876000
934400
992600
1051200
1109600
1168000
1226400
1284800
1343200
1401600
1460000
1518400
1576800
1635200
1693600
1752000
1810400
1868800
1927200
1985600
2044000
2102400
2160800
2219200
2277600
2336000
