# Supplementary material for: Intrinsic DNA curvature in trypanosomes
Source: BMC Res Notes. 2017 Nov 9;10:585. doi: 10.1186/s13104-017-2908-y (PMC5679330; doi:10.1186/s13104-017-2908-y)
Supplement: Supplementary file 4 — Additional file 4: Figure S2. Graphical representation of sequence dependent curvature in T. brucei chromosomes. The chromosome number is depicted at the top of each page. Upper panel: Bar plots of chromosome positions with an IC value greater than 13 degrees per helical turn. Middle panel: Bar plots of chromosome positions with an RIIC value greater than the selected cutoff. Lower panel: both chromosome DNA strands are depicted in grey, overlaid with CDS features shown in blue. Features labeled as ncRNA, snRNA or snoRNAs are shown in green. tRNAs are shown in red. Assembly gaps are shown in brown. Subtelomeric VSG clusters are underlined. [file 13104_2017_2908_MOESM4_ESM.pdf]

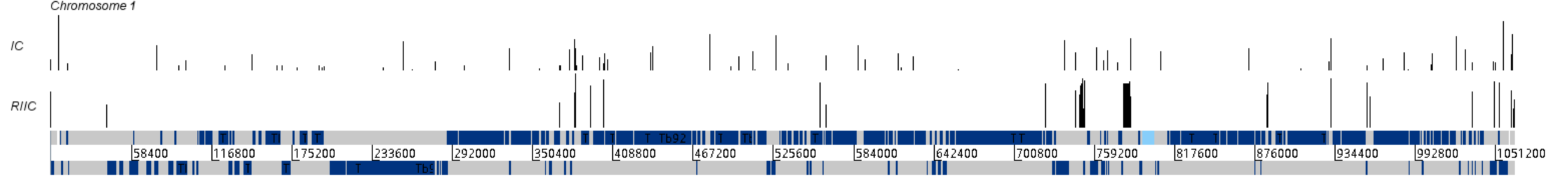
Chromosome 1
IC
RIIC
T
T
T
T
Tb92
58400
116800
175200
233600
292000
350400
408800
467200
525600
584000
642400
700800
759200
817600
876000
934400
992800
1051200

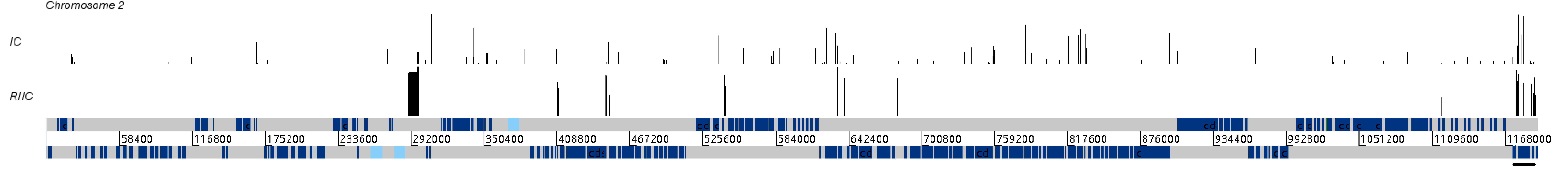

Chromosome 2
IC
RIIC
58400
116800
175200
233600
292000
350400
408800
467200
525600
584000
642400
700800
759200
817600
876000
934400
992800
1051200
1109600
1168000

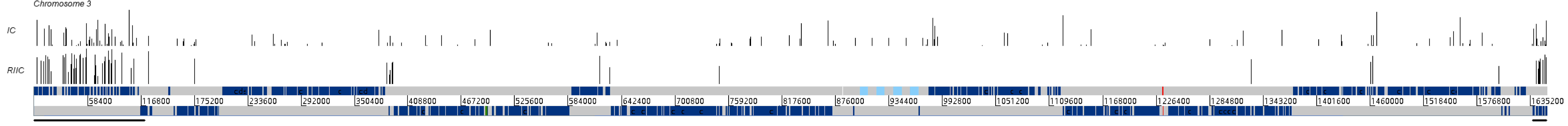
Chromosome 3
IC
RIIC
58400
116800
175200
233600
292000
350400
408800
467200
525600
584000
642400
700800
759200
817600
876000
934400
992800
1051200
1109600
1168000
1226400
1284800
1343200
1401600
1460000
1518400
1576800
1635200

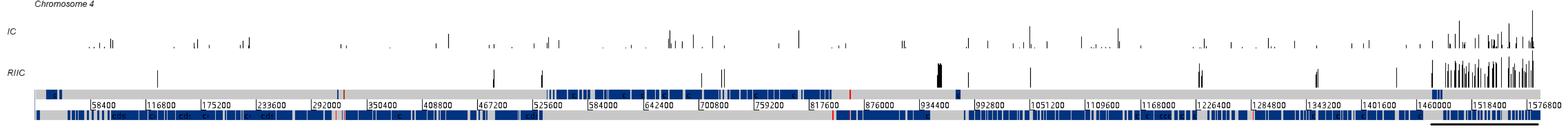

Chromosome 4
IC
RIIC
58400
116800
175200
233600
292000
350400
408800
467200
525600
584000
642400
700800
759200
817600
876000
934400
992800
1051200
1109600
1168000
1226400
1284800
1343200
1401600
1460000
1518400
1576800

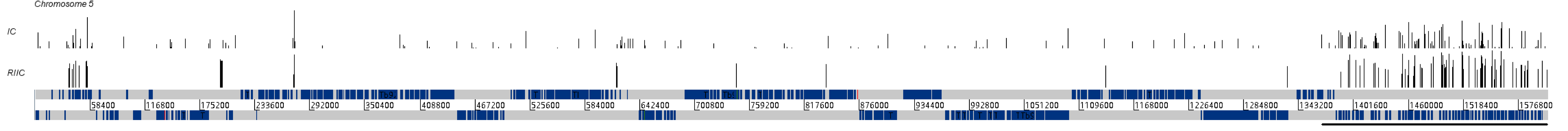
Chromosome 5
IC
RIIC
58400
116800
175200
233600
292000
350400
408800
467200
525600
584000
642400
700800
759200
817600
876000
934400
992800
1051200
1109600
1168000
1226400
1284800
1343200
1401600
1460000
1518400
1576800

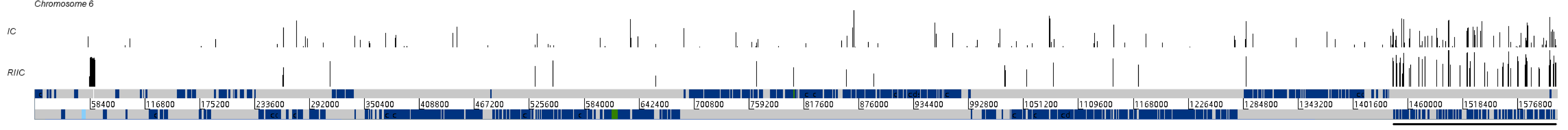
Chromosome 6
IC
RIIC
58400
116800
175200
233600
292000
350400
408800
467200
525600
584000
642400
700800
759200
817600
876000
934400
992800
1051200
1109600
1168000
1226400
1284800
1343200
1401600
1460000
1518400
1576800

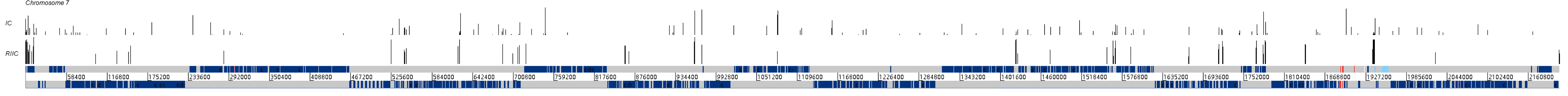
Chromosome 7
IC
RIIC
58400
116800
175200
233600
292000
350400
408800
467200
525600
584000
642400
700800
759200
817600
876000
934400
992800
1051200
1109600
1168000
1226400
1284800
1343200
1401600
1460000
1518400
1576800
1635200
1693600
1752000
1810400
1868800
1927200
1985600
2044000
2102400
2160800

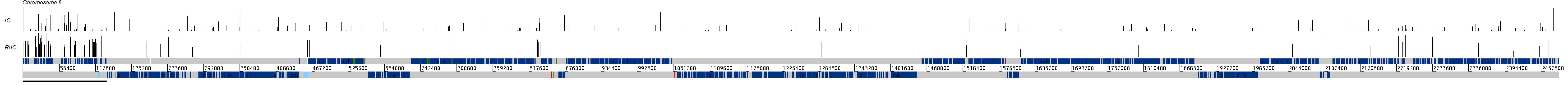
Chromosome 8
IC
RIIC
58400
116800
175200
233600
292000
350400
408800
467200
525600
584000
642400
700800
759200
817600
876000
934400
992800
1051200
1109600
1168000
1226400
1284800
1343200
1401600
1460000
1518400
1576800
1635200
1693600
1752000
1810400
1868800
1927200
1985600
2044000
2102400
2160800
2219200
2277600
2336000
2394400
2452800

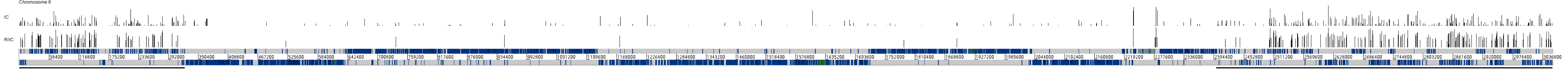

Chromosome 9
IC
RIIC
58400
116800
175200
233600
292000
350400
408800
467200
525600
584000
642400
700800
759200
817600
876000
934400
992800
1051200
1109600
1168000
1226400
1284800
1343200
1460000
1518400
1576800
1635200
1693600
1752000
1810400
1868800
1927200
1985600
2044000
2102400
2160800
2219200
2277600
2336000
2394400
2452800
2511200
2569600
2628000
2686400
2744800
2803200
2861600
2920000
2978400
3036800

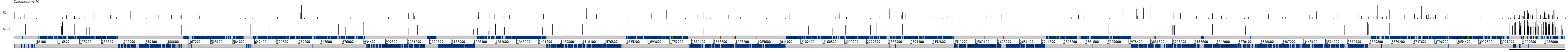
Chromosome 10
IC
RIIC

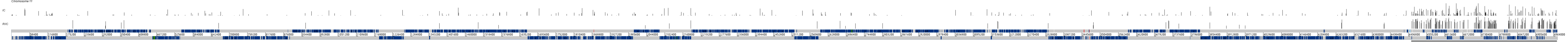
Chromosome 11
IC
RVC
