# Supplementary material for: Intrinsic DNA curvature in trypanosomes
Source: BMC Res Notes. 2017 Nov 9;10:585. doi: 10.1186/s13104-017-2908-y (PMC5679330; doi:10.1186/s13104-017-2908-y)
Supplement: Supplementary file 5 — Additional file 5: Figure S3. Graphical representation of transcription markers’ signals and sequence dependent curvature RIIC score in T. brucei. For each T. brucei chromosome, the graphical representation of regions with RIIC value greater than the selected cutoff (lower panel) are shown above the schematic representation of both chromosome DNA strands depicted in grey, overlaid with CDS features shown in blue. Modified histone locations (H4K10ac) from [17] and base J from [12] are indicated as following: regions associated to H4K10ac but not associated with base J (*); regions associated to H4K10ac and also with base J (!); regions associated with base J and not with H4K10ac (+). Features labeled as ncRNA, snRNA or snoRNAs are shown in green. tRNAs are shown in red. Assembly gaps are shown in brown. [file 13104_2017_2908_MOESM5_ESM.pdf]

Chromosome 1

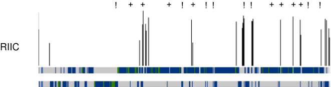

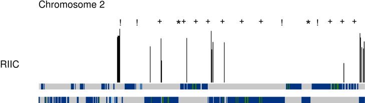

Chromosome 2
RIIC

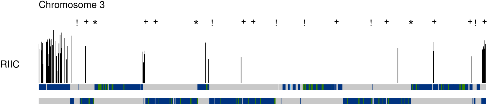

Chromosome 3
RIIC

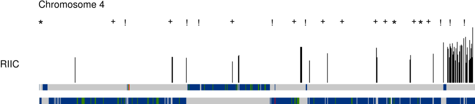
Chromosome 4
RIIC

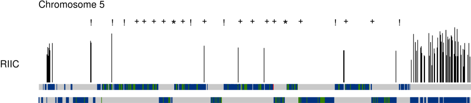
Chromosome 5
RIIC

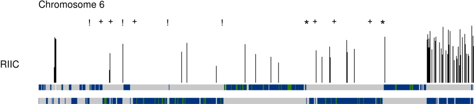
Chromosome 6
RIIC
! + + ! + ! ! * + + + *

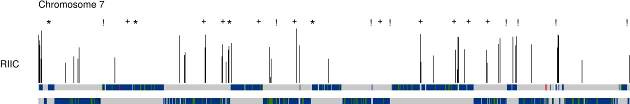
Chromosome 7
RIIC

Chromosome 8

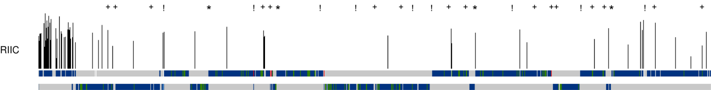

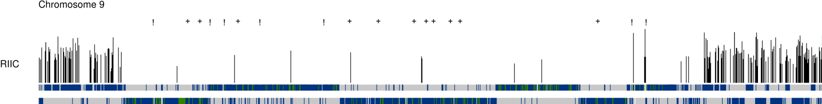
Chromosome 9
RIIC

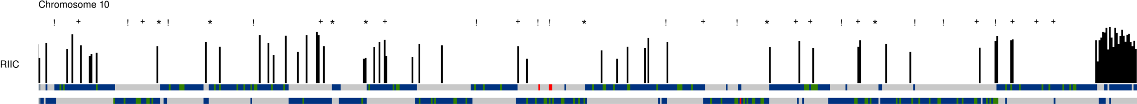

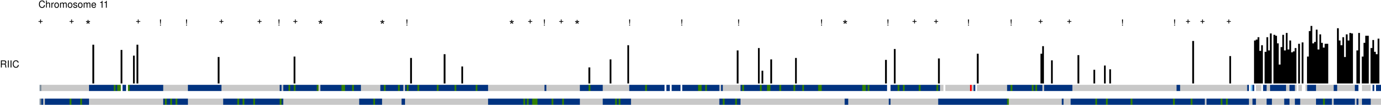
Chromosome 11
RIIC
